# Phase Behavior and Conformational Asymmetry near the Comb-to-Bottlebrush Transition in Linear-Brush Block Copolymers

**DOI:** 10.1021/acs.macromol.3c02180

**Published:** 2024-02-20

**Authors:** Regina J. Sánchez-Leija, Joshua A. Mysona, Juan J. de Pablo, Paul F. Nealey

**Affiliations:** † Materials Science Division, Argonne National Laboratory, 9700 S Cass Avenue, Lemont, Illinois 60439, United States; ‡ Pritzker School of Molecular Engineering, the University of Chicago, 5640 S Ellis Avenue, Chicago, Illinois 60637, United States

## Abstract

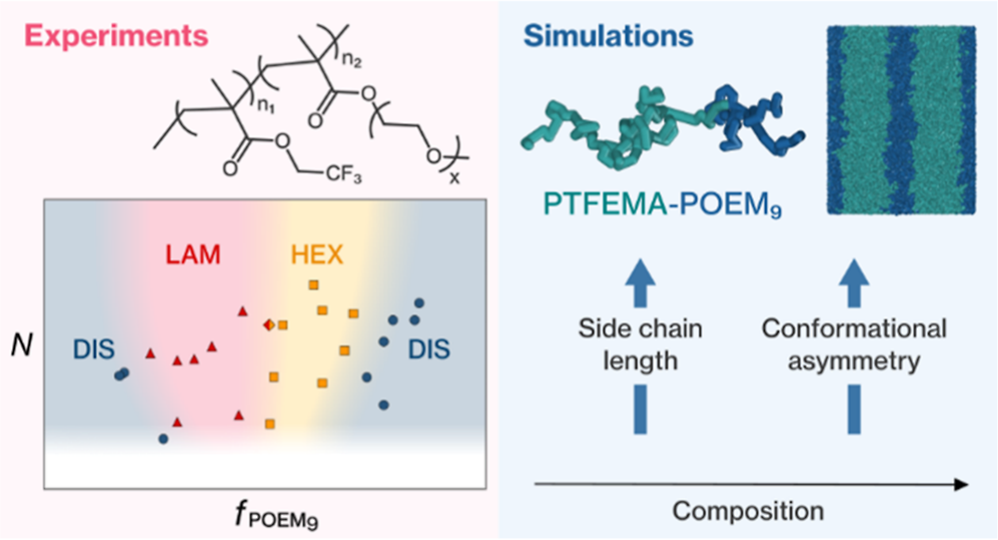

This study explores how conformational asymmetry influences the bulk phase behavior of linear-brush block copolymers. We synthesized 60 diblock copolymers composed of poly(trifluoroethyl methacrylate) as the linear block and poly[oligo(ethylene glycol) methyl ether methacrylate] as the brush block, varying the molecular weight, composition, and side-chain length to introduce different degrees of conformational asymmetry. Using small-angle X-ray scattering, we determined the morphology and phase diagrams for three different side-chain length systems, mainly observing lamellar and cylindrical phases. Increasing the side-chain length of the brush block from three to nine ethylene oxide units introduces sufficient asymmetry between the blocks to alter the phase behavior, shifting the lamellar-to-cylindrical transitions toward lower brush block compositions and transitioning the brush block from the dense comb-like regime to the bottlebrush regime. Coarse-grained simulations support our experimental observations and provide a mapping between the composition and conformational asymmetry. A comparison of our findings to strong stretching theory across multiple phase boundary predictions confirms the transition between the dense comb-like regime and the bottlebrush regime.

## Introduction

Block copolymers (BCPs) are versatile materials, as they exhibit self-assembly into ordered nanostructures that combine the properties of their constituent blocks. In linear AB BCPs, the volume fraction of the A component (*f*_A_) and segregation strength (χ*N*), where χ is the Flory–Huggins interaction parameter and *N* the total degree of polymerization, determine the nanoscale equilibrium morphology and feature size by quantifying the competition between the energetic stretching penalties of the blocks.^1−3^ In the case of nonlinear AB BCPs where one block is linear and the other exhibits a branched topology, conformational asymmetry must also be considered, as it can have a profound influence on the phase boundaries.^4,5^

Branched polymer architectures, such as bottlebrushes, barbed wires, comb-like and Y-shaped structures offer a pathway to thermodynamically tune feature size and phase behavior by introducing a high degree of conformational asymmetry and creating curved interfaces at symmetric volume fractions during self-assembly.^6,7^ The parameter  is commonly used as a measure of conformational asymmetry, accounting for differences in molecular architecture and in how the blocks fill space^8,9^ (β is the ratio of segment length to segment volume of each block). For conformationally symmetric AB BCPs, ε is equal to 1. While conformational asymmetry can alter the phase behavior of linear BCPs, accessible values of ε are typically limited and close to 1.

Conformationally asymmetric BCP structures can enable larger or smaller domain spacings than those of analogous linear AB BCPs,^10,11^ offering compositional flexibility surpassing that of conformationally symmetric BCPs. However, moving from linear AB BCPs to more complex macromolecular architectures complicates the mapping of the design parameter space to the phase separation behavior. Experimental efforts have therefore focused on investigating the phase behavior as a function of additional variables, such as side-chain length and graft density of the branched blocks, to determine morphology and feature size and build the corresponding phase diagrams.^6,12−16^

Theoretical studies have attempted to predict the magnitude and direction of the deflection of the phase boundaries for a variety of comb and bottlebrush polymer architectures. In particular, strong stretching theory (SST) provides boundaries for the order–order transitions between lamellar, cylindrical and sphere-forming phases for linear-bottlebrush BCPs.^17,18^ These studies characterize the order–order transition as a function of two parameters: *f*_B_, the volume fraction of the bottlebrush block, and , a parameter coupling the asymmetry between blocks and the morphology of the bottlebrush block, where η_A_ and η_B_ are architecture-dependent topological ratios that quantify the relative energetic contributions of the blocks. For linear polymers, the parameter η_A_ is defined as 1, while the combined parameter  for copolymers with a linear block A and bottlebrush block B is
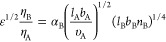
1where *l*_A_ is the monomer A length, *b*_A_ is the Kuhn length, υ_A_ is the monomer volume, *n*_B_ is the bottlebrush side-chain length, and α is a numerical prefactor, which must be adjusted and fixed according to the position of one of the phase boundaries. Alternatively, for a linear-comb architecture, the scaling behavior of the asymmetry is quantitatively different and given by
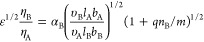
2where *q* is the number of side chains per branch, *n* is the side-chain length, and *m* is the spacer degree of polymerization.^18^ We note that in an ideal test of the scaling theory, α would be used to determine the boundaries across multiple phase transitions for both comb-like and bottlebrush-like polymers, but achieving this comparison in experiments can be difficult.

In addition to SST, self-consistent field theory (SCFT) has been used to predict the deflection of phase boundaries caused by asymmetry and to identify the gyroid window for certain BCP systems, with some studies showing good agreement between experiments and theory.^5,19−24^ Other field-theoretic efforts have focused on investigating the phase behavior of statistical bottlebrushes and establishing a universal phase diagram based upon the relation between asymmetry and bottlebrush architectural parameters.^25,26^ In these studies, which examined linear-brush BCPs with varying degrees of asymmetry across both the comb-like and bottlebrush regimes, the authors did not observe a transition between the comb-like and bottlebrush scaling of the phase diagram as described by Zhulina *et al.*(18) Instead, they found that the polymers behaved as combs in all cases and attributed the absence of such a transition to the inherent limitations of SCFT.

The inherent structural complexity of branched BCPs makes particle-based simulations computationally expensive,^27,28^ as they require long equilibration times for side chains with a large number of repeat units. While numerous simulation studies have examined the melt state of bottlebrushes with a focus on their conformal properties,^29−31^ BCP systems require extended times to self-assemble. Consequently, coarse-grained simulations are preferred over atomistic simulations for studying self-assembly behavior, although accurately mapping coarse-grained simulations to phase behavior is also difficult because the necessary parameter spaces ε, *f*, and χ*N* must be indirectly inferred.

Several attempts have been made to circumvent these issues. One well-developed approach for simulating the self-assembly of bottlebrush polymers utilizes an implicit potential for the polymer side chain, which reduces the computational intensity.^32,33^ This approach shows good agreement with the experimental structure factors but has yet to be tested against scaling theories for phase boundaries. Other methods for predicting phase behavior employ distinct bonded interactions between the backbone and the side chain to accelerate simulation.^34^ In studies on the self-assembly of bottlebrush polymers in solution, the solvent has been either modeled implicitly^35^ or explicitly in combination with dissipative particle dynamics type models.^36−38^ None of these efforts, however, have been used to compare the scaling in asymmetry between combs and bottlebrushes, as theoretically proposed by Zhulina *et al.*(18)

While experimental and simulation studies have explored the phase behavior of linear-brush BCP architectures with varying degrees of conformational asymmetry, quantification of the extent of overlap between side chains as a function of side-chain length is frequently absent in these investigations. In linear-brush BCPs such as linear-comb and linear-bottlebrush, the nature of the architecture is given by the graft density and side-chain length. These two variables enable systematic control of conformation and the degree of overlap and entanglement with neighboring molecules,^30,31,39^ which has significant implications for the physical properties of polymers in both melt and solution states.^40^ Precise control of graft density, side-chain length, molecular weight, and tailored functional groups can be achieved through controlled/living polymerization techniques [*i.e.*, atom transfer radical polymerization, reversible addition–fragmentation chain transfer (RAFT) polymerization, nitroxide-mediated radical polymerization (NMP)], anionic polymerization, ring-opening polymerization, ring-opening metathesis polymerization, and “click” coupling reactions.^41−43^ These branched polymers have potential applications in areas such as nanolithography,^44−47^ biomedicine,^48−50^ energy storage,^51−53^ and beyond, emphasizing the importance of gaining a comprehensive understanding of their behavior.^54^

Here, we investigate how conformational asymmetry affects the bulk phase behavior of linear-brush BCPs consisting of poly(trifluoroethyl methacrylate) (PTFEMA) as the linear block and poly[oligo(ethylene glycol) methyl ether methacrylate] (POEM) as the brush block. We use RAFT polymerization to synthesize a library of 60 PTFEMA-*b*-POEM copolymers with varying degrees of polymerization, composition and POEM side-chain length. In this study, we alter the degree of conformational asymmetry between the blocks by changing the side-chain length and systematically study the phase behavior. BCP morphology and the corresponding phase diagrams are determined for three PTFEMA-*b*-POEM_*x*_ copolymer systems [*x* = 3, 5, or 9: number of ethylene oxide (EO) units in the POEM side chain] using small-angle X-ray scattering (SAXS). Coarse-grained simulations support our experimental observations and enable a more complete mapping of the relationship between the composition and the conformational asymmetry. We test whether we observe a transition between comb- and bottlebrush-like behavior in simulation and verify the results of SST for both linear-brush architectures. A comparison of our findings to SST predictions further demonstrates the potential for using the theory for the design of highly asymmetric BCP systems.

## Results and Discussion

### Synthesis of the Linear-Brush Diblock Copolymers

**Scheme 1:**
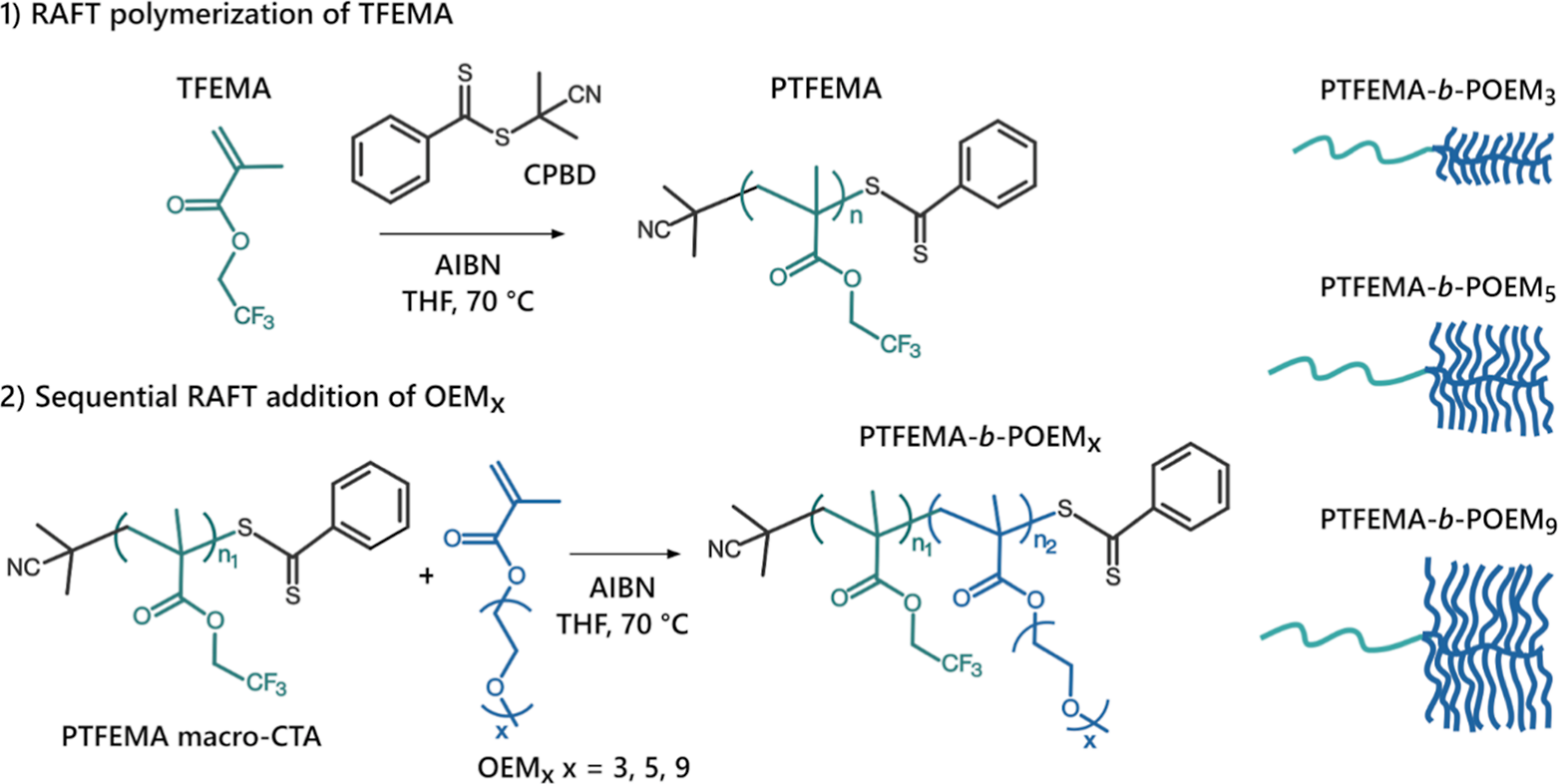
RAFT Polymerization Scheme for the Synthesis of PTFEMA-*b*-POEM_*x*_ Copolymers

The synthesis of PTFEMA-*b*-POEM_*x*_ copolymers involved two steps (Scheme 1). First, we conducted RAFT polymerization of 2,2,2-trifluoroethyl methacrylate (TFEMA) using 2-cyano-2-propyl benzodithioate (CPBD) as the RAFT agent to obtain a series of PTFEMA with average molecular weights (*M*_*n*_) ranging from 5.2 to 24.7 kg mol^–1^ (Figure S1). The RAFT polymerization of TFEMA exhibited first-order kinetic behavior, characterized by a linear relationship between the polymerization rate and the logarithm of the monomer concentration over time (Figure S2a). As the polymerization proceeded, *M*_*n*_ increased linearly with monomer conversion, while dispersity (*D̵*) remained narrow (Figure S2b). These observations indicate a well-controlled RAFT polymerization.^55,56^

Second, we performed the sequential polymerization of oligo(ethylene glycol) methyl ether methacrylate (OEM_*x*_, *x* = 3, 5, or 9: number of EO units) using the PTFEMA homopolymers synthesized in step 1 as RAFT agents. Following this protocol, we prepared two sets of diblock copolymers with similar *M*_*n*_ (around 20 and 30 kg mol^–1^) and different compositions for each side-chain length (Figures S6, S8, and S10). In a later section, we discuss where the POEM side-chain length becomes sufficient to be considered bottlebrush-like. Based upon the theoretical framework by Dobrynin and co-workers,^31^ we believe this transition occurs roughly at *x* = 5.

The chemical composition, *M*_*n*_, and dispersity (*D̵*) of the resulting polymers were determined using ^1^H NMR, ^19^F NMR, and SEC. Detailed structural characterization of the full set of polymers is provided in the Supporting Information. The ^1^H NMR and ^19^F NMR spectra of PTFEMA (Figures S3 and S4) and PTFEMA-*b*-POEM_*x*_ copolymers (Figures S5, S7 and S9) exhibited the characteristic chemical shifts corresponding to the protons and fluorine atoms present in the chemical structures of these polymers.^57−61^ The chemical compositions of the diblock copolymers were determined from the molar ratios calculated from the ^1^H NMR peak integrals of the −CH_2_–CF_3_ (δ = 4.34 ppm: *s*, 2H) and −C(O)–O–CH_2_– (δ = 4.08 ppm: *s*, 2H) signals ascribed to the PTFEMA and POEM_*x*_ blocks, respectively. Table 1 lists the compositions in mass fraction of the brush block (*x*_POEM*x*_), as well as the corresponding *M*_*n*_ and *D̵* values for a representative set of PTFEMA-*b*-POEM_*x*_ copolymers with approximately 30 kg mol^–1^. Dispersity ranged from 1.13 to 1.33, within the typical values obtained for well-controlled RAFT polymerization.

**Table 1:** Characteristics of the PTFEMA-*b*-POEM_*x*_ Copolymers

BCP	*M*_*n*_ (kg mol^–^^1^)	*D̵*	*x*_POEMx_^a^	*N*_POEMx_^b^	*N*_total_^b^	*f*_POEM_^b^	morphology^c^
PTFEMA-*b*-POEM_3_	31.3	1.33	0.21	79	319	0.25	hexagonal
	30.7	1.33	0.24	88	315	0.28	hex + lam
	29.3	1.33	0.30	106	305	0.35	lamellar
	27.8	1.21	0.40	134	296	0.45	lamellar
	28.1	1.19	0.57	193	310	0.62	hexagonal
	29.0	1.15	0.69	241	328	0.73	hexagonal
	28.9	1.15	0.82	285	335	0.85	disordered
PTFEMA-*b*-POEM_5_	27.1	1.27	0.09	30	269	0.11	disordered
	25.7	1.19	0.23	72	264	0.27	hexagonal
	27.7	1.20	0.26	87	286	0.31	hex + lam
	27.5	1.20	0.28	93	286	0.33	lamellar
	26.7	1.19	0.37	120	283	0.42	lamellar
	24.1	1.14	0.54	158	266	0.59	hexagonal
	26.0	1.15	0.80	252	303	0.83	disordered
PTFEMA-*b*-POEM_9_	27.4	1.24	0.15	51	277	0.18	disordered
	29.1	1.26	0.20	73	299	0.24	lamellar
	28.0	1.22	0.25	87	291	0.30	lamellar
	27.9	1.21	0.29	101	293	0.34	lamellar
	29.1	1.18	0.32	116	308	0.38	lamellar
	30.4	1.19	0.45	170	332	0.51	lam + hex
	30.2	1.16	0.48	180	333	0.54	hexagonal
	31.2	1.18	0.57	221	351	0.63	hexagonal
	30.3	1.18	0.64	241	347	0.70	hexagonal
	28.8	1.13	0.74	266	339	0.79	disordered
	30.0	1.14	0.82	306	359	0.85	disordered

a Calculated from the molar ratio of both blocks determined by ^1^H NMR.

b Calculated using a 118 Å^3^ reference volume and densities of ρ(PTFEMA) = 1.45 g/cm^3^, ρ(POEM_3_) = 1.17 g/cm^3^ (25 °C), ρ(POEM_5_) = 1.16 g/cm^3^ (25 °C), and ρ(POEM_9_) = 1.13 g/cm^3^ (25 °C).

c Determined using SAXS.

The total degree of polymerization (*N*) and volume fraction (*f*) of the blocks were calculated using the standard definitions for monomer volume equivalents (see Supporting Information). We assumed a density of 1.45 g cm^–3^ for PTFEMA, based on previous reports,^62,63^ and estimated the densities for POEM_3_, POEM_5_, and POEM_9_ using the van Krevelen group contribution method^64,65^ (1.17, 1.16, and 1.13 g cm^–3^, respectively). Table 1 shows the *N* and *f* values for a set of PTFEMA-*b*-POEM_*x*_ copolymers.

### Experimental Phase Diagrams and Impact of Side-Chain Length

**Figure 1:**
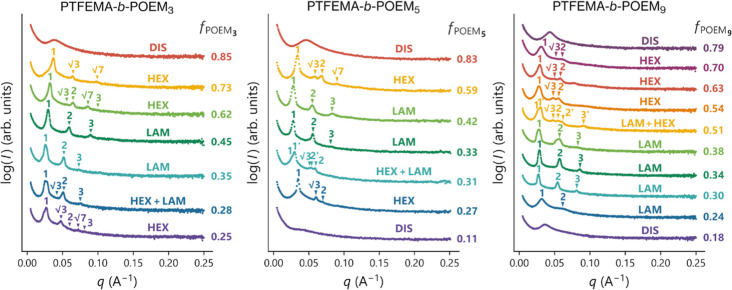
Room temperature SAXS patterns for a representative set of the PTFEMA-*b*-POEM_*x*_ copolymers (approximately 30 kg mol^–1^) as a function of the volume fraction of the POEM_*x*_ block (*f*_POEMx_). DIS, HEX, and LAM denote disordered, hexagonal (cylindrical), and lamellar phases, respectively.

**Figure 2:**
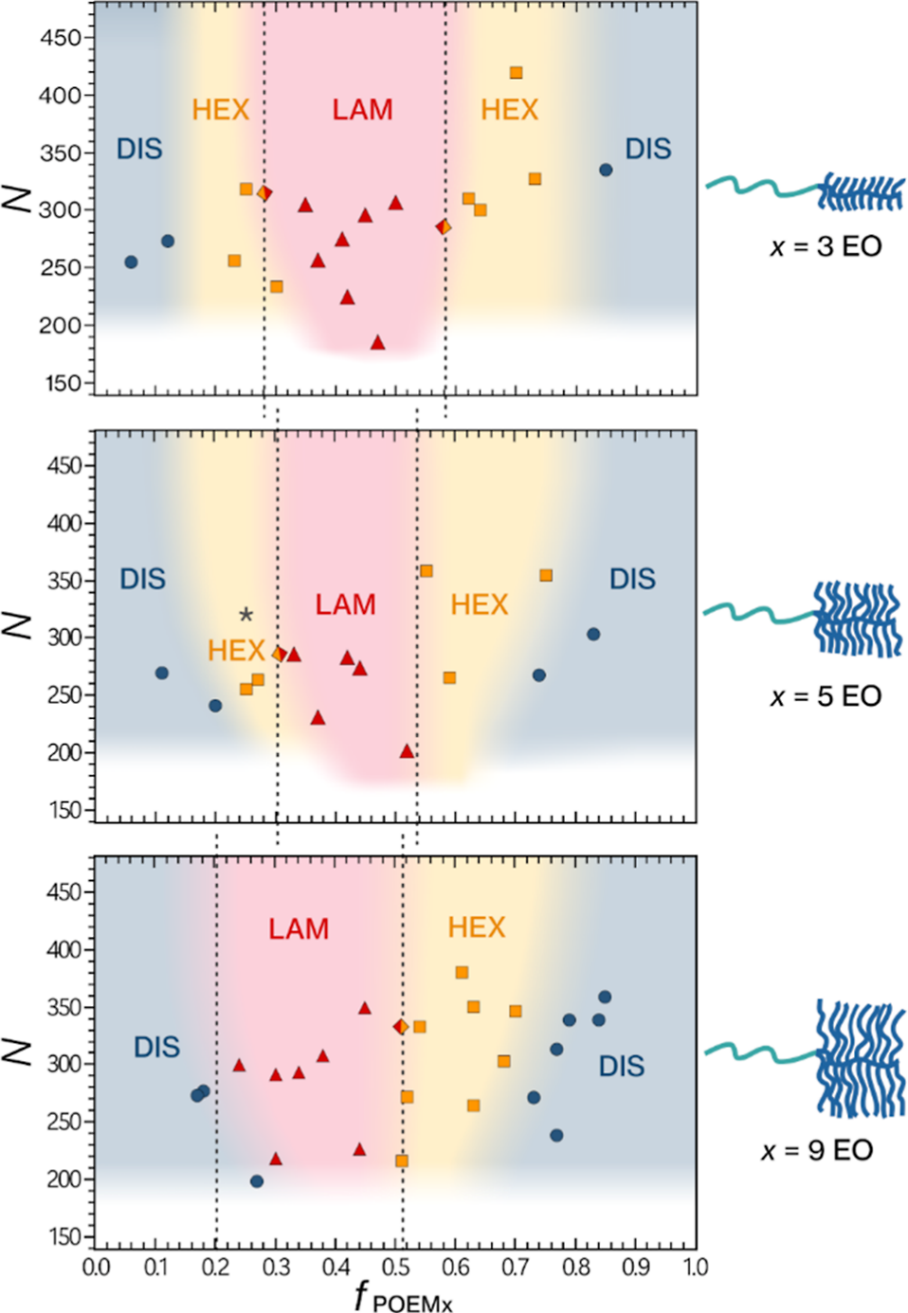
Phase diagrams determined by SAXS for the PTFEMA-*b*-POEM_*x*_ copolymers as a function of volume fraction of the POEM_*x*_ block (*f*_POEMx_) and the total degree of polymerization (*N*); *x* denotes the number or EO units in the side chain of the brush block. The star symbol in the PTFEMA-*b*-POEM_5_ phase diagram denotes the possible coexistence of hexagonal and gyroid phases. Colors used for phase regions and dashed lines are exclusively intended as visual aids and should not be interpreted as delineating actual phase boundaries.

We employed SAXS to further characterize the diblock copolymers on samples annealed for 20 h at 150 °C to favor self-assembly. Figure 1 displays the SAXS patterns obtained at room temperature for the PTFEMA-*b*-POEM_*x*_ copolymers listed in Table 1. The SAXS patterns acquired for all the diblock copolymers, along with the peak indexing, are provided in the Supporting Information (Figures S11–S13). Analysis of the SAXS data enabled us to map the bulk morphologies as functions of *f* and *N* and construct the phase diagrams illustrated in Figure 2. Notably, *N* is the vertical axis in our phase diagrams rather than segregation strength (χ*N*); there is currently no theoretical framework for χ that accurately accounts for the entropic restrictions caused by the presence of side chains in branched BCP architectures.^14^

We identified disordered, hexagonal (...), and lamellar () phases from the corresponding SAXS patterns. Multiple sets of scattering reflections were also observed at several POEM volume fractions (*f*_POEM*x*_) across the three linear-brush systems, indicating phase coexistence near the phase boundaries. The presence of sharp primary and higher-order peaks in the SAXS patterns of samples exhibiting ordered phases suggests strong phase separation and long-range order. We found significant differences between the linear-brush systems as the POEM side-chain length increases from 3 to 9 EO units. The PTFEMA-*b*-POEM_3_ phase diagram closely resembles the conventional phase diagram for a linear AB BCP, with the lamellar region 0.26 ≲ *f*_POEM3_ ≲ 0.60. In PTFEMA-*b*-POEM_3_, the polymer segments are not substantially different in the ratio of the statistical segment length to segment volume and do not introduce sufficient conformational asymmetry to significantly shift the phase boundaries.

Increasing the side-chain length by 2 EO units in the PTFEMA-*b*-POEM_5_ system shows a slight narrowing of the lamellar region to *f*_POEM5_ ∼ 0.54. When *f*_POEM5_ = 0.25 and *N* = 319 (star symbol in the phase diagram), indexing of the SAXS pattern suggests the coexistence of hexagonally packed cylinders and the gyroid phase (Figure S12, entry # 14). We note that the (220) diffraction peak associated with the gyroid phase and characterized by  exhibits very low intensity in the corresponding SAXS pattern. This peak appears as a shoulder within the primary peak, making it difficult to assert with absolute certainty the presence of the gyroid phase in the PTFEMA-*b*-POEM_5_ phase diagram.

In contrast, the PTFEMA-*b*-POEM_9_ phase diagram exhibits significant asymmetry and deviates from that of conventional linear AB BCPs. The lamellar phase region shifts to 0.18 ≲ *f*_POEM9_ ≲ 0.5, while the cylindrical phase region spans 0.5 ≲ *f*_POEM9_ ≲ 0.8. Linear AB BCPs exhibit cylindrical morphology at a minority block fraction of *f* < 0.3. The side-chain length of 9 EO units introduces a high degree of conformational asymmetry between the blocks, which changes the relative stretching penalties of the blocks and consequently affects phase behavior. Specifically, when the linear blocks are the minority, the system exhibits a thermodynamic preference for spontaneous curvature toward the linear blocks, resulting in the formation of hexagonally packed PTFEMA cylinders in a POEM_9_ matrix.

The influence of conformational asymmetry on the phase diagram of diverse branched diblock copolymer architectures have been investigated through SCFT in the strong segregation regime.^4,5^ Our experimental findings align with a recent SCFT computational study conducted by Park *et al.*,^21^ which focused on investigating the stability of double gyroid network phases in bottlebrush diblock copolymer melts. For linear-bottlebrush architectures, these SCFT calculations predict that when the linear blocks constitute the minority, increasing the side-chain length of the bottlebrush block leads to a shift in the phase boundaries toward higher compositions of the linear block.

Additionally, Liberman *et al.* conducted comprehensive experimental studies on norbornene-based linear-bottlebrush BCPs, systematically varying polarity of the linear block and side-chain length of the bottlebrush block.^14,15^ They observed hexagonally packed cylinders, double gyroid and lamellae phases in a set of 223 diblock copolymers with volume fractions of the linear block ranging from 0.30 to 0.70, total degrees of polymerization ranging from 30 to 140, and side-chain lengths from 1 to 9 ethylene-*alt*-propylene repeat units.^15^ These studies show that increasing the conformational asymmetry induces phase coexistence and shifts the phase boundaries toward lower compositions of the linear block, altering the double gyroid compositional window. As conformational asymmetry increases, the maximum values and ranges of *N* associated with the gyroid-forming copolymer decrease while the χ parameter increases, resulting in limited or no access to the order–disorder transition (ODT). The absence of gyroid phases in our phase diagrams may result from the narrowing of the compositional window derived from asymmetry, making it difficult to experimentally access that region, and the limited or no access to the ODT, as PTFEMA-*b*-POEM_*x*_ copolymers likely exhibit a high χ value.

### Assessment of POEM_*x*_ Bottlebrush-like Behavior

The scaling model of graft polymers introduced by Dobrynin classifies comb-like and bottlebrush architectures in a melt based on the crowding parameter, Φ.^30,31^ This parameter quantifies the degree of interpenetration between side chains and the backbones of neighboring macromolecules by comparing the volume occupied by the side chains of a single polymer to the side chain pervaded volume. The transition from an unstretched state to the bottlebrush state occurs at Φ ≈ 1, although this transition is relatively broad. In the comb regime (Φ < 1), the side chains and backbones of adjacent macromolecules interpenetrate, causing comb-like architectures to behave similar to linear chain backbones. Conversely, in the bottlebrush regime (Φ ≥ 1), steric repulsion between side chains prevents interpenetration between macromolecules.^30,31^ Consequently, proper classification of polymers as comb-like or bottlebrush-like is critical because the calculation of asymmetry between these two regimes is different. To calculate Φ, we follow the work of Dobrynin and first estimate the volume pervaded by the side chain as
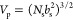
3where *N*_s_ is the side-chain degree of polymerization and *b* is the statistical segment length.

The approximate amount of the pervaded volume filled by the chain itself is the sum of the backbone volume and side chain volume of a chain with a length equal to the backbone. The corresponding number of backbone monomers is given by
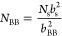
4where *N*_BB_ is the number of backbone monomers and *b*_BB_ is the unperturbed statistical segment length of the backbone. The total volume is thus
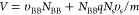
5where *υ*_BB_ and υ_s_ are the relevant monomer volumes. Note that up to this point, we have not assumed equivalent volume or flexibility between the backbone and side chains, which is particularly relevant in the case of POEM, where the PEO-like side chains and the PMMA-like backbone have drastically different volumes and flexibilities. Combining eqs 3–5, the expression of Φ is given by
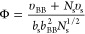
6which reduces to the expression given by Dobrynin^30^ if it is assumed the volumes and statistical segment lengths are equal between the backbone and the side chain.

**Figure 3:**
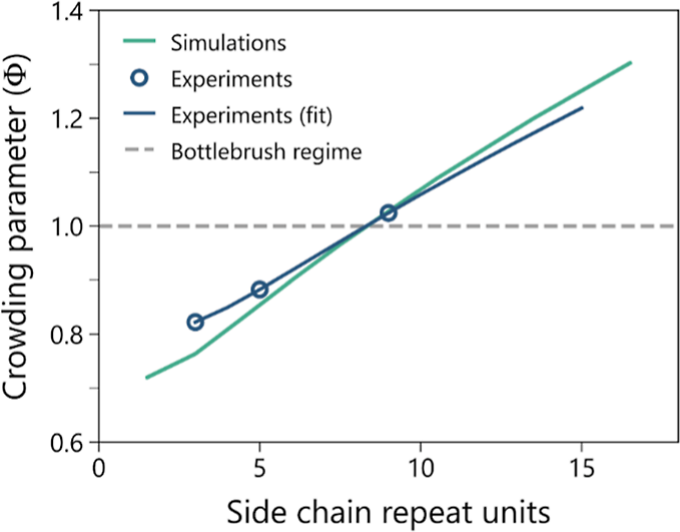
Crowding parameter Φ *vs* side-chain length for POEM. Circles represent the experimental systems chosen, and the green solid line represents the simulated systems. The blue solid line represents the value of Φ for other POEM side-chain lengths based on the experimental results. The dashed line is Φ = 1, bottlebrush regime. Φ values were calculated from eq 6. For the POEM system, *b*_s_ was assumed to be equal to 0.56 nm, *b*_BB_ = 0.65 nm, υ_BB_ = 0.14 nm^3^, and υ_s_ = 0.067 nm^3^. Discrepancy between simulation and experimental points is due to the discretization difference in the coarse-grained simulation.

Results from these calculations for the experimental and simulated systems are presented in Figure 3. Surprisingly, despite the low degree of polymerization of the side chains in the examined POEM blocks, we find both POEM_5_ and POEM_9_ to be at the threshold of transitioning into well-defined bottlebrush structures with Φ values very close to 1. While for POEM_5_ Φ is slightly below 1 (∼0.9), this value is sufficient for the polymer to begin exhibiting bottlebrush-like behavior in the first regime, in which crowding of side chains begins to stretch the polymer backbone. In contrast, Φ is < 1 for POEM_3_, which indicates that the polymer is in the comb regime. The simulated system also follows this trend, and brush blocks with side-chain lengths of five simulation beads or greater can reasonably be classified as bottlebrush-like. This is relevant as numerous experimental studies on bottlebrush architectures lack characterization of the comb-to-bottlebrush transition and often report the synthesis of longer brushes, usually involving side chains comprising tens or more monomer units.^41,54^ Our observations suggest that brushes of shorter length may be enough for certain systems to exhibit bottlebrush-like behavior. These findings emphasize the important role that side-chain length plays in the design of BCPs with branched architectures. It not only influences phase behavior but also governs the comb-to-bottlebrush transition, which has significant implications for the physical properties (*e.g.*, rheological and mechanical)^40,66^ and processability of these polymers in both the melt and solution states.

### Comparison of Experiments with Theoretical Order–Order Transitions

Subsequently, we aimed to compare the experimentally observed order–order phase transitions for the three linear-bottlebrush BCP systems with those predicted by SST. These order–order transitions are described by the asymmetry parameter  and the volume fraction of the brush *f*_B_, as opposed to those used in the previous phase diagrams described by *N* and *f*_B_. This comparison attempts to establish the relationship between the polymer architecture and morphology. With larger values of , the brush-like block becomes more asymmetric, and the tendency for the interface to curve away from the brush-like block increases.

To compare between theory and experiments, we compute the parameter  for each architecture pairing. However, this requires assigning a value to each constant in eq 1. While constants such as *l*_A_ and *b*_A_ in this equation have reliable literature values, the parameter α is a numerical prefactor not easy to measure experimentally. We followed the methodology presented by Zhulina *et al.*,^18^ which utilizes the placement of one order–order transition to determine the numerical prefactor and further calculates it for the POEM_9_ system using the lamella-to-cylinder transition where the major volume fraction is bottlebrush. This transition and system best reflect the phase behavior because the majority bottlebrush lamellar-to-cylindrical transition is the deepest in the ordered phase with the longest blocks and thus closest to the conditions assumed in SST. Using this prefactor alongside the η_B_ scaling, we then determine the values of  for the other two systems.

**Figure 4:**
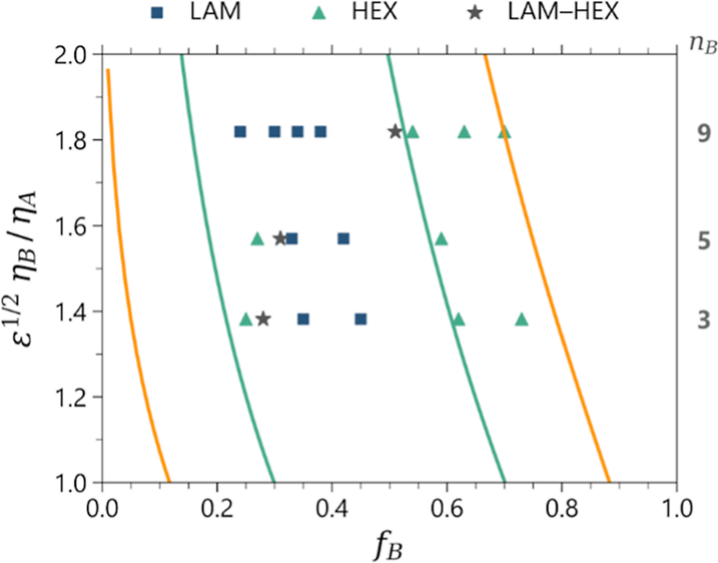
Asymmetry phase diagram for the experimental PTFEMA-b-POEM_*x*_ systems listed in Table 1. The *x* axis is the relative volume fraction of bottlebrush block *f*_B_, while the *y* axis is the asymmetry parameter. *n*_B_ denotes the number of repeat units in the side chains. Yellow and green solid lines represent the cylinder-to-lamella and lamella-to-cylinder transitions predicted by the SST.

Figure 4 plots the order–order transitions as discussed previously as well as morphology as a function of the POEM volume fraction for the series of PTFEMA-*b*-POEMx copolymers listed in Table 1. The results are best understood by comparing the majority bottlebrush lamella-to-cylinder transition separately from the majority linear lamella-to-cylinder transition. The lamella-to-cylinder transition in the majority bottlebrush region shows excellent agreement between SST and the experimental results, and both the POEM_5_ and POEM_3_ BCP systems scale with *n*_B_^1/4^ based on SST, even though POEM_3_ is not a bottlebrush polymer. In contrast, the lamella-to-cylinder transition in the minority bottlebrush region occurs earlier than would be otherwise predicted by SST. It is noteworthy that changing how we determined the parameter  alone cannot resolve this discrepancy, as the width between these points is determined solely by *f*_B_, which is known with high accuracy. Any shift large enough to correct the issue would also introduce a correspondingly large error in the majority of the bottlebrush transition. Instead, we hypothesize that this phase diagram deviation arises from our system not being in the strong-stretching segregation regime, as assumed in the theory. The absence of a further spherical phase and the presence of disorder in the experimental phase diagrams may suggest that we are approaching an ODT. If so, this suggests that in the experimental system, the degree of segregation is weaker than that required by SST. While there does not exist in the literature a study that combines the effects of conformational asymmetry and variable segregation strength, we suspect that the behavior may be similar to the symmetric case, where weaker segregation leads to a narrowing of the lamellar window.

### Comparison of Simulations with Theoretical Order–Order Transitions

Simulation methods enabled the exploration of the phase diagram for a variety of asymmetries to further investigate the effects of asymmetry and order–order transitions in the PTFEMA-*b*-POEM_*x*_ systems. For all simulations, the diblock copolymer consisted of 120 total beads distributed between the linear block and the brush-like block. We changed the number of chains in the brush-like block while keeping the number of total beads constant, following a similar approach as Wessels and Jayaraman.^36^ This resulted in a significant variation in the volumetric degree of polymerization in the brush-like block, as in our experiments. Details on the architectures studied can be found in the Supporting Information (Tables S9 and S10).

**Figure 5:**
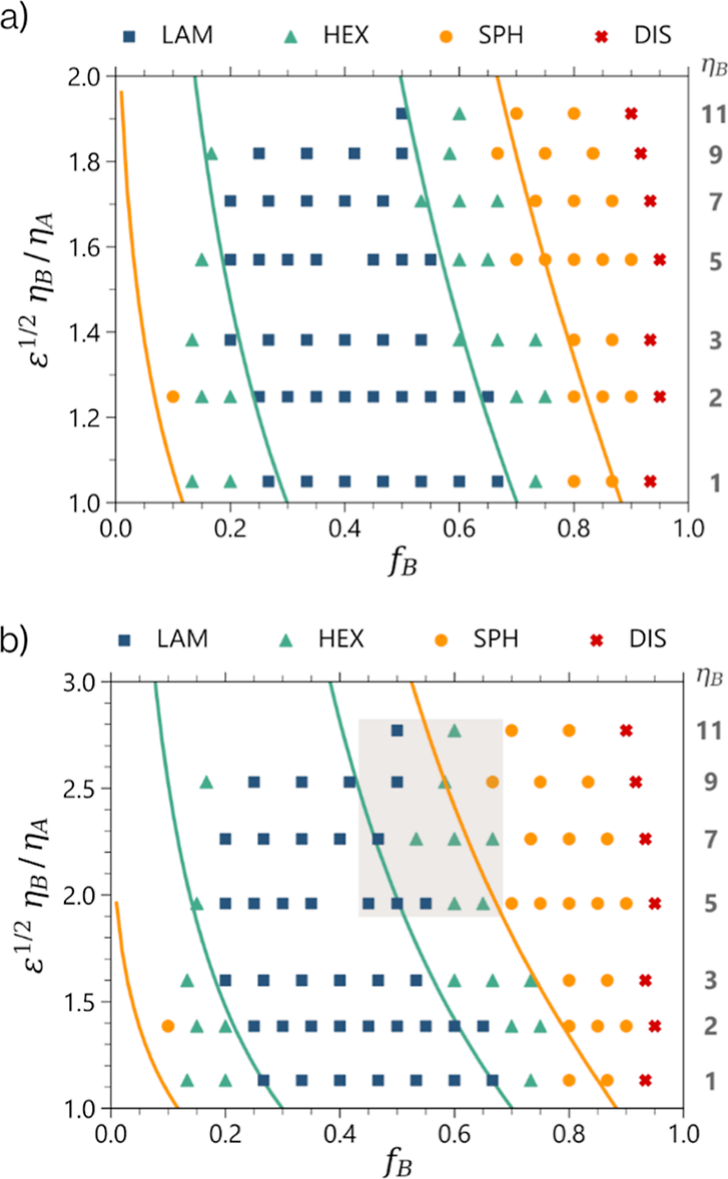
Simulated asymmetry phase diagrams for the PTFEMA-*b*-POEM_*x*_ systems determined by using (a) bottlebrush and (b) comb scaling methods. The *x* axis is the relative volume fraction of bottlebrush block *f*_B_, while the *y* axis is the asymmetry parameter. *n*_B_ denotes the number of beads in the side chains. Yellow and green solid lines represent the cylinder-to-lamella and lamella-to-cylinder transitions predicted by the SST. Note that these lines are the same in both plots, but the asymmetry parameter was calculated using either eq 1 (bottlebrush scaling) or eq 2 (comb scaling), respectively. The shaded area highlights the discrepancies observed in the lamella-to-cylinder transition for the comb-like scaling between the simulation data and SST for BCPs with side chains of 5 or more monomer units.

The asymmetry parameter  can be calculated alongside the unperturbed linear parameters for the simulated BCP system based on simulations of the bottlebrush homopolymer for various side-chain lengths, where α is used as an adjustable parameter. The resulting phase diagram is displayed in Figure 5a. Intermediate phases are difficult to determine without resorting to free energy methods and have not been resolved in this study. This is especially true for the lamellar phase and the majority of bottlebrush cylinder phases, which feature a substantial number of perforated lamellar-like structures. The theoretical phase boundaries were calculated as described by Zhulina *et al.*(17) and are depicted as solid lines.

The comparison of the experimental and simulated phase diagrams affords several observations. First, contrary to previous literature,^14,15,21^ we surprisingly do not find a gyroid phase, which agrees with our experimental results. The gyroid phase is notoriously difficult to manifest in particle-based simulations due to the sensitive requirements of matching the unit cell dimension; even if a stable gyroid phase window exists, it is unlikely to be encountered. Additionally, χ is relatively high in the simulated system, resulting in a narrow gyroid region that can be easily overlooked. Second, we observe spherical phases at higher *f*_B_ compositions in the simulated phase diagrams in contrast to the experimental ones. This discrepancy might be related to the fact that the spherical phase window is very narrow before the system disorders and may have been missed in the experimental systems, where the resolution of *f*_B_ is lower. Moreover, the energy barrier could be large such that, at the temperature samples were measured, it would be difficult for the spherical phases to nucleate. Lastly, the phase boundaries shift to lower values of *f*_B_, in agreement with the SST, as the large bottlebrush monomer force increases the interfacial curvature to accommodate their conformations. The SST predictions for the order–order transitions show remarkable agreement with the simulation results in the transitions observed between each ordered phase. Moreover, it is worthwhile noting that even though all the polymers with Φ close to one fit well using eq 1 for the linear-bottlebrush BCP architectures, we see some deviation for the shorter side chain polymers, *n*_s_ equal to one and two.

The theoretical model proposed by Zhulina *et al.*(18) predicts a difference in scaling of the asymmetry parameter between a comb-like and a more bottlebrush-like polymer architecture. To investigate this further, we calculated the asymmetry parameter for linear-comb BCP architectures with varying side-chain length according to eq 2.^18^ Upon plotting the phase behavior using eq 2 in Figure 5b, we observe a good fit for 1, 2, and 3 bead side chains. However, as side-chain length increases, the trend predicted by eq 2 overestimates the asymmetry of the bottlebrush polymers (1/2 scaling for combs versus 1/4 scaling for bottlebrushes). This overestimate results in an incorrect prediction for the lamella-to-cylinder transition for the more asymmetric bottlebrush polymers.

From this analysis, we conclude that BCPs with lower side-chain lengths are best described as linear-comb, while those with longer side-chain lengths exhibit proper bottlebrush behavior, in agreement with the findings by Zhulina.^18^ We believe that this behavior suggests that there does exist a transition in the phase behavior when moving from comb-like to bottlebrush-like side chains.

## Conclusions

In this study, we investigated the phase behavior of a family of PTFEMA-*b*-POEM_*x*_ copolymers. Alongside experiments, we performed simulations on a coarse-grained BCP system with a wide range of side-chain lengths to further explore the phase diagram as a function of conformational asymmetry. The experimental and simulated systems agree well with the SST, with the latter showing stronger agreement. We attribute discrepancies in the experimental system, particularly in the lamella-to-cylinder transition in the minority bottlebrush region, to its proximity to the ODT. Nevertheless, our study demonstrates that SST can be used to design highly asymmetric BCP systems that self-assemble into predictable morphologies, even at unconventional volume fractions.

A key aspect of our study is the comparison of the experimental and simulation data to SST predictions for the corresponding phase boundaries as the system transitions from a comb-like block to a bottlebrush block architecture. We find evidence that suggests that this transition occurs within the range of side-chain lengths studied. Our findings align with the argument of Fredrickson that the absence of predictions of bottlebrush behavior in their SCFT stems from modeling the system using a mean-field approach and highlight the importance of treating these two branched architectures differently when studied from a theoretical standpoint.

The close agreement between the simulation work in this study and the theory is quite remarkable. The use of a simulated polymer system with identical monomer volumes and bonds between monomers enabled a clean test of the SST and precluded speculation on certain asymmetries. By delineating the regime for which SST works, we show that this theoretical framework can be used to guide the future design of these systems to accelerate material development.

## Experimental Section

### Materials

TFEMA (99%), OEM_*x*_ (*x* = 3, 5, or 9; *M*_*n*_ = 232.27 g mol^–1^, *M*_*n*_ = 300 g mol^–1^, and *M*_*n*_ = 500 g mol^–1^, respectively), CPBD (>97%), azobisisobutyronitrile (AIBN), and deuterated chloroform [CDCl_3_, 99.8 atom % D, 0.03% (v/v) TMS] were purchased from Sigma-Aldrich. Tetrahydrofuran (THF, HPLC grade) and hexanes were purchased from Fisher Chemical. TFEMA and OEM_*x*_ monomers were purified before use to remove inhibitors by being passed through a column of basic alumina. AIBN was recrystallized in ethanol before use.

### Synthesis of the PTFEMA Macro-Chain Transfer Agents

PTFEMA macro-chain transfer agents (macro-CTA) were synthesized *via* RAFT polymerization of TFEMA in THF using CPBD as the chain transfer agent and AIBN as the initiator. Number average molecular weights (*M*_*n*_) of 10, 25, 35, 45, and 50 kg mol^–1^ were targeted to obtain a series of homopolymers with varying *M*_*n*_ while keeping TFEMA monomer conversion well below 75% to reduce prevalence of dead polymer chains.

To synthesize PTFEMA (*M*_*n*_ target = 10 kg mol^–1^), the following procedure was used. TFEMA (4 g, 23.8 mmol), CPBD (88.5 mg, 0.4 mmol), AIBN (6.6 mg, 0.04 mmol), and THF (2.985 mL, ∼ 8 M) were stirred in a reaction tube and purged with dry N_2_ for 20 min. Multiple tubes with the same reagent quantities were prepared and placed in a carousel reactor for conducting parallel kinetics experiments and polymerizations. The reaction temperature was maintained at 70 °C, and the reflux carousel head was cooled by using water at 5 °C. Polymerization reactions were quenched by rapidly immersing the tubes in liquid N_2_ at different times, followed by exposure to air. Aliquots were taken from the crude solutions to assess monomer conversion. The crude solutions were precipitated into excess hexanes, followed by redissolution in THF and precipitation in hexanes two more times. The resulting polymers were dried at 50 °C under vacuum overnight. The same experimental protocol and conditions were followed when targeting *M*_*n*_ of 25, 35, 45, and 50 kg mol^–1^.

### Synthesis of the PTFEMA-*b*-POEM_*x*_ Copolymers

PTFEMA-*b*-POEM_*x*_ (*x* = 3, 5, or 9: number of EO side-chain units) copolymer series of ∼20 and 30 kg mol^–1^ were synthesized *via* RAFT polymerization of OEM_*x*_ monomers with PTFEMA as the RAFT agent and AIBN as the initiator. For instance, PTFEMA-*b*-POEM_9_ (Table S4, entry # 6) was synthesized by mixing PTFEMA # 4 (*M*_*n*_ = 7.5 kg mol^–1^, 0.5 g, 67 μmol), OEM_9_ (1.55 g, 3.1 mmol), AIBN (1.1 mg, 6.7 μmol), and THF (2.83 mL, [OEM_9_] ∼ 1 M) in a reaction tube and purged with dry N_2_ for 20 min. The reaction temperature was 70 °C and the reflux carousel head was cooled using water at 5 °C. Polymerization reactions were quenched by rapidly immersing the tubes in liquid N_2_ at different time points, followed by exposure to air. The crude solutions were precipitated into excess hexanes, followed by redissolution in THF and precipitation in hexanes two more times. The resulting BCPs were dried at 50 °C under a vacuum overnight. PTFEMA-*b*-POEM_5_ and PTFEMA-*b*-POEM_3_ copolymers were synthesized following the same synthesis and purification protocols. ^1^H NMR spectra of representative sets of PTFEMA-*b*-POEM_*x*_ are shown in Figures S5, S7, and S9. GPC traces for the complete series of the diblock copolymers are shown in Figures S6, S8, and S10, while a summary of the reaction parameters employed and their corresponding TFEMA mass fraction (*x*_TFEMA_), *M*_*n*_, and *D̵* are listed in Tables S2–S4.

### Proton (^1^H) and Fluorine (^19^F) Nuclear Magnetic Spectroscopy

^1^H and ^19^F NMR experiments were performed on a 400 MHz Bruker AVANCE III HD Nanobay spectrometer equipped with an iProbe SmartProbe. Spectra were acquired at 25 °C using CDCl_3_ as a solvent and a polymer concentration between 10 and 15 mg mL^–1^. For ^1^H NMR spectra acquisition, 64 scans were collected at a relaxation delay time of 5 s to determine conversion and chemical composition; while for ^19^F NMR spectra, 32 scans were collected at a relaxation delay time of 1 s. Proton chemical shifts were referenced to tetramethylsilane (TMS), and all data were processed with Mnova NMR software.

### Size Exclusion Chromatography

Size exclusion chromatography (SEC) measurements were conducted on a Wyatt/Shimadzu instrument using THF as an eluent at a flow rate of 1 mL min^–1^. Polymer solutions were prepared at a concentration of 4 mg mL^–1^ and passed through 0.20 μm poly(tetrafluoroethylene) filters. Separation was achieved using two Agilent PLgel 5 μm Mix-D columns maintained at 27 °C after injection of 35 μL of polymer solution. Number average molecular weight (*M*_*n*_) and dispersity (*D̵*) were determined by comparison with polystyrene standards.

### Small-Angle X-ray Scattering

SAXS measurements were performed using the SAXSLAB GANESHA instrument at the University of Chicago X-ray Facility. Prior to the experiments, dry diblock copolymer samples were annealed in bulk under vacuum at 150 °C for 20 h and then slowly cooled to ambient temperature. Data was collected at ambient temperature for 30 min.

### Simulation Details

The simulation utilized is a simple dissipative particle dynamic-like soft model. The bonded interactions between beads are governed by a potential
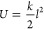
7with *k* equal to 4.0. The nonbonded potential between beads is governed by
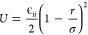
8where the factor σ is equal to one and ϵ_*ii*_ is equal to 25*k*_B_*T*. For unlike beads, the interaction is defined by ϵ_*ij*_ = ϵ_*ii*_ + α_*ij*_. For the simulations present here, α_AB_ has been set to 1.5*k*_B_*T*, which controls the free energy of mixing, with a higher value of α corresponding to a higher free energy of mixing and thus decreased contact between the A and B type beads. Molecular dynamics simulations are carried out in the *NPT* ensemble with *T* = 1.0 and *P* = 20.249 to achieve a bead density of approximately ρ = 3.0/σ^3^. In this ensemble, only the *y* and *z* dimensions are coupled to each other, while *x* is allowed to freely fluctuate to allow the box dimensionality to fluctuate and relax to be commensurate with the lowest free energy phase. All calculations were carried out using HOOMD-blue v2.9.3.^67^ All simulations were allowed to relax over 500τ_60_, where τ_60_ is the time for the linear part of the diblock to relax when it is composed of 60 beads or *f*_A_ equal to 0.50.

For each linear block brush block architecture, chains were randomly placed and then allowed to equilibrate as listed above. In all cases, *f*_A_ was varied from approximately 0.2 to 0.8, stopping at *N*_B_ equal to 3, where *N*_B_ is the degree of polymerization. The side-chain length was denoted as *n*_B_ and varied from 1 all the way to 9. The bond constants were held the same in both blocks, and so the only changes in conformal asymmetry originate from the different monomer side-chain lengths and conformal changes due to stretching the bottlebrush. The asymmetries in this system are designed to span the range of those encompassed by the experimental system.

## Supporting Information Available

^1^H NMR and ^19^F NMR spectra, SEC traces, SAXS patterns, molecular characteristics of the linear-bottlebrush BCPs, density estimation by the van Krevelen group contribution method, parameters setting for SCFT from experimental data, SCFT parameters, and simulation details (PDF)
