# Supplementary material for: Phase Behavior and Conformational Asymmetry near the Comb-to-Bottlebrush Transition in Linear-Brush Block Copolymers
Source: Macromolecules. 2024 Feb 20;57(5):2019–29. doi: 10.1021/acs.macromol.3c02180 (PMC10938885; doi:10.1021/acs.macromol.3c02180)
Supplement: Supplementary file 1 — ma3c02180_si_001.pdf [file ma3c02180_si_001.pdf]

Supporting Information

Phase Behavior and Conformational Asymmetry Near the Comb-to-Bottlebrush Transition in Linear-Brush Block Copolymers

Regina J. Sánchez-Leija,[1,2] Joshua A. Mysona,[1,2]

Juan. J. de Pablo,[1,2] Paul F. Nealey[1,2*]

[1]Pritzker School of Molecular Engineering, University of Chicago, 5640 S. Ellis Ave, Chicago, IL 60637, United States

[2]Materials Science Division, Argonne National Laboratory, 9700 South Cass Ave, Lemont, IL 60439, United States

*nealey@uchicago.edu

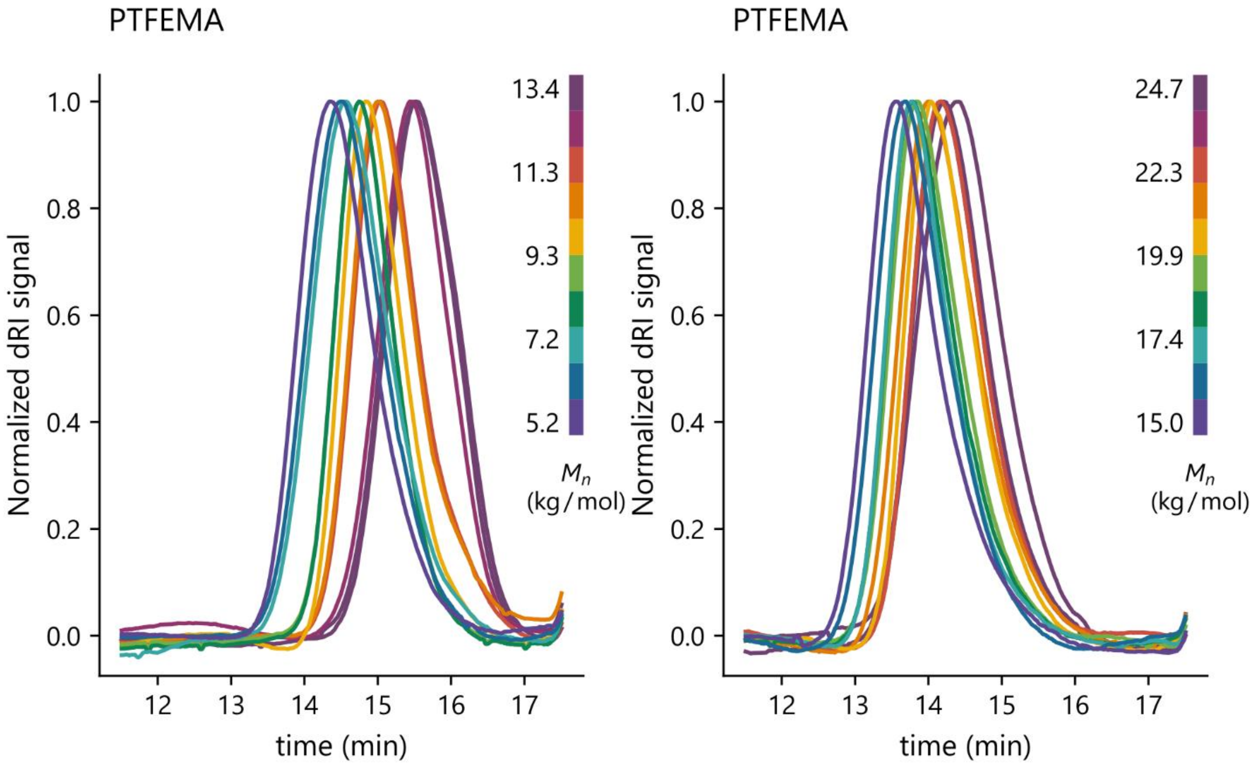

Figure S1. SEC traces of the full set of PTFEMA homopolymers. M_n values ranged from 5.2 kg mol^{-1} to 24.7 kg mol^{-1}.

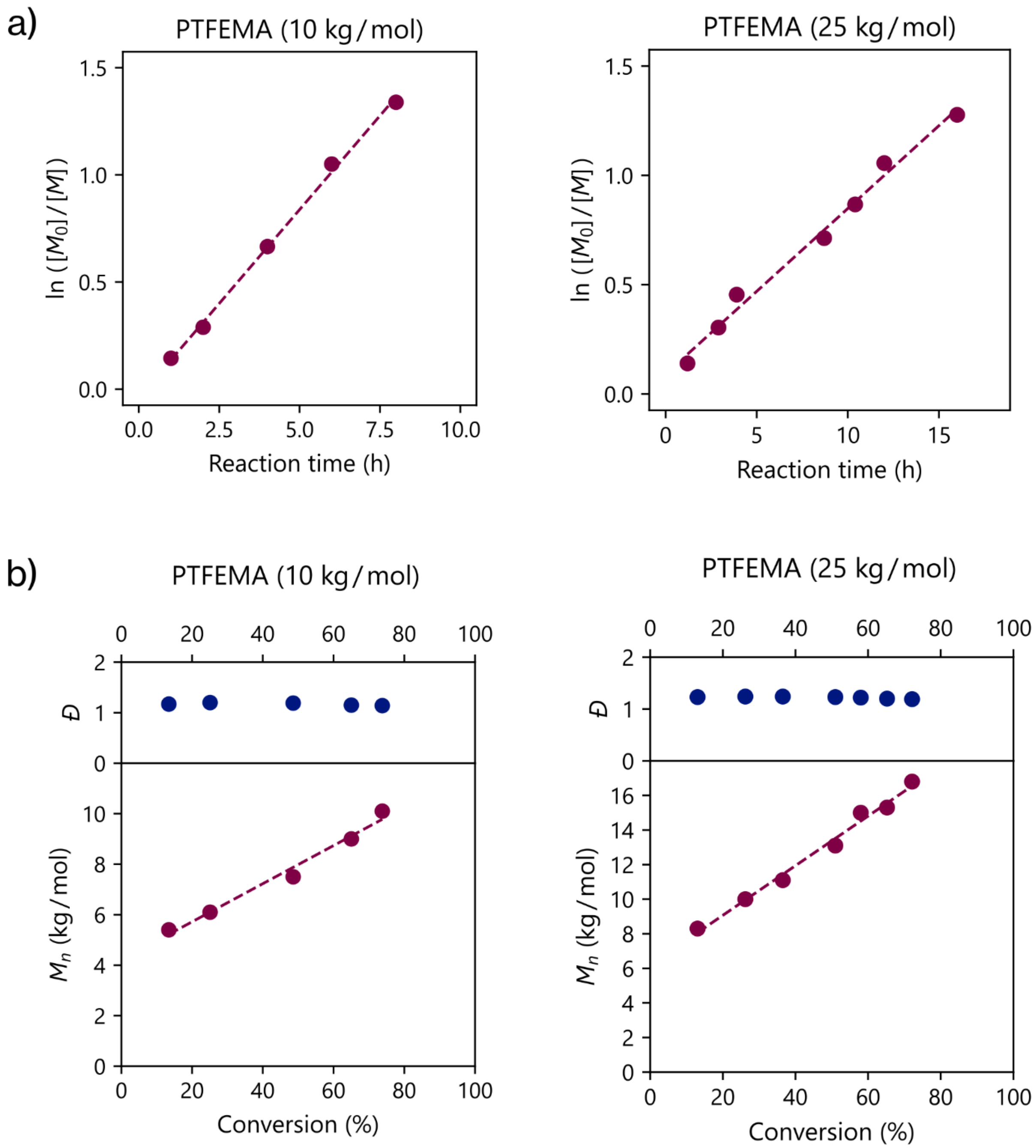

Figure S2. RAFT polymerization of TFEMA using AIBN as initiator and CPBD as chain transfer agent. (a) Evolution of experimental M_n and $Đ$ as function of monomer conversion. (b) ln([M]$_0$/[M]) as function of reaction time, with [M]$_0$ and [M] being the concentrations of monomers at time zero and t, respectively.

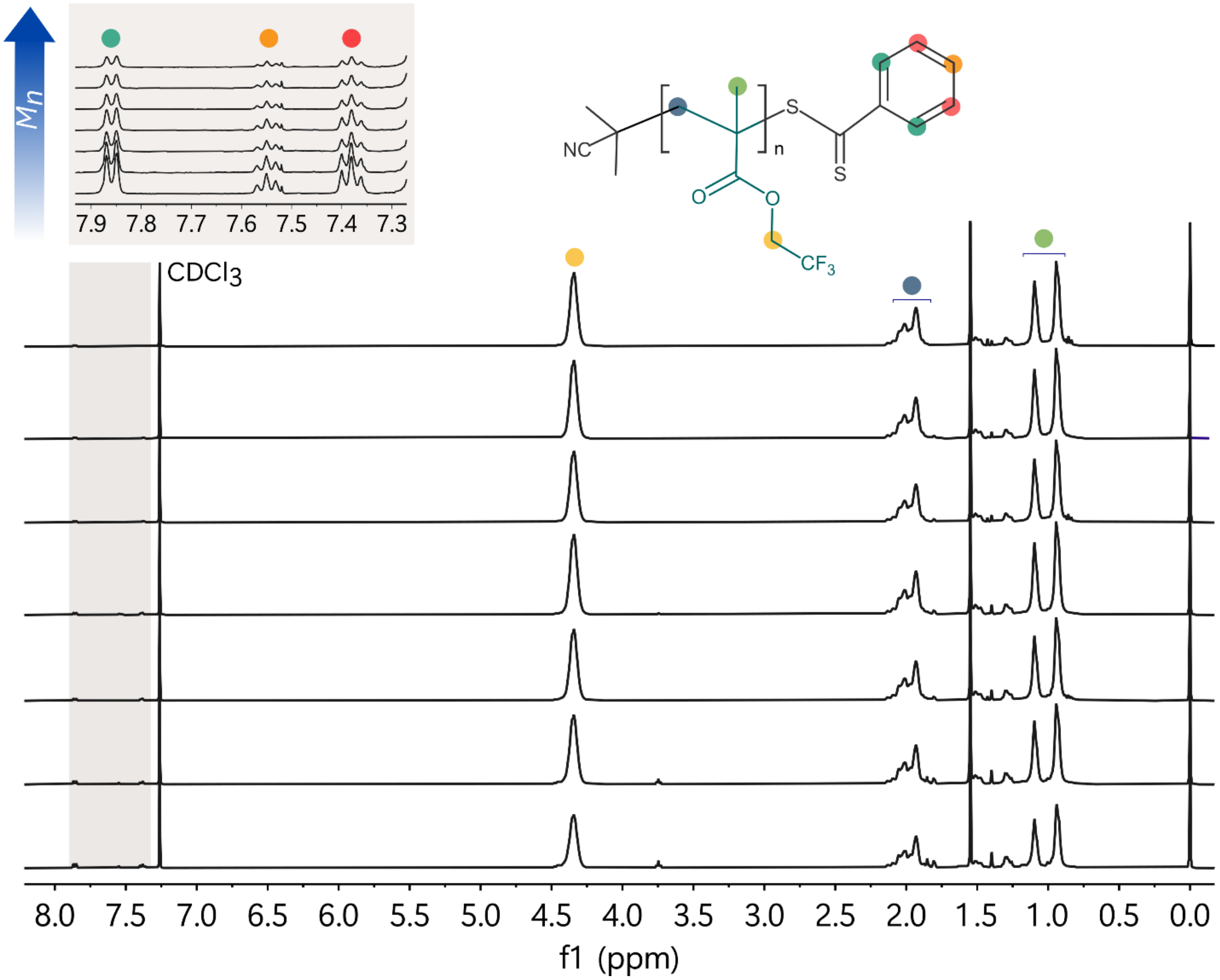

Figure S3. ^{1}H NMR (400 MHz, $CDCl_3$) spectra of PTFEMA macro-chain transfer agents (macro-CTA). δ (ppm): 0.80–1.20 (*m*, 3H, –C**H$_3$**), 1.80–2.20 (*m*, 2H, –C**H$_2$**–), 4.34 (*s*, 2H, –C**H$_2$**–CF_3), 7.34–7.42 (*t*, 2H, protons of aromatic ring), 7.5–7.6 (*t*, 2H, protons of aromatic ring) 7.83–7.90 (*d*, 2H, protons of aromatic ring).[1,2]

TFEMA conversion was determined from the ^{1}H NMR peak integrals (*I*) of PTFEMA at δ = 4.34 ppm (2H, −CH_2−CF_3), and TFEMA at δ = 6.23 ppm and 5.70 ppm (2H, H_2C=C<) according to the following expression:

$$X_{TFEMA} = 1 - \frac{I[-CH_2 - \mathrm{CF_3}\ (\mathrm{PTFEMA})]}{I[-CH_2 - \mathrm{CF_3}\ (\mathrm{PTFEMA})] + I[H_2\mathrm{C} = \mathrm{C} < (\mathrm{TFEMA})]}$$

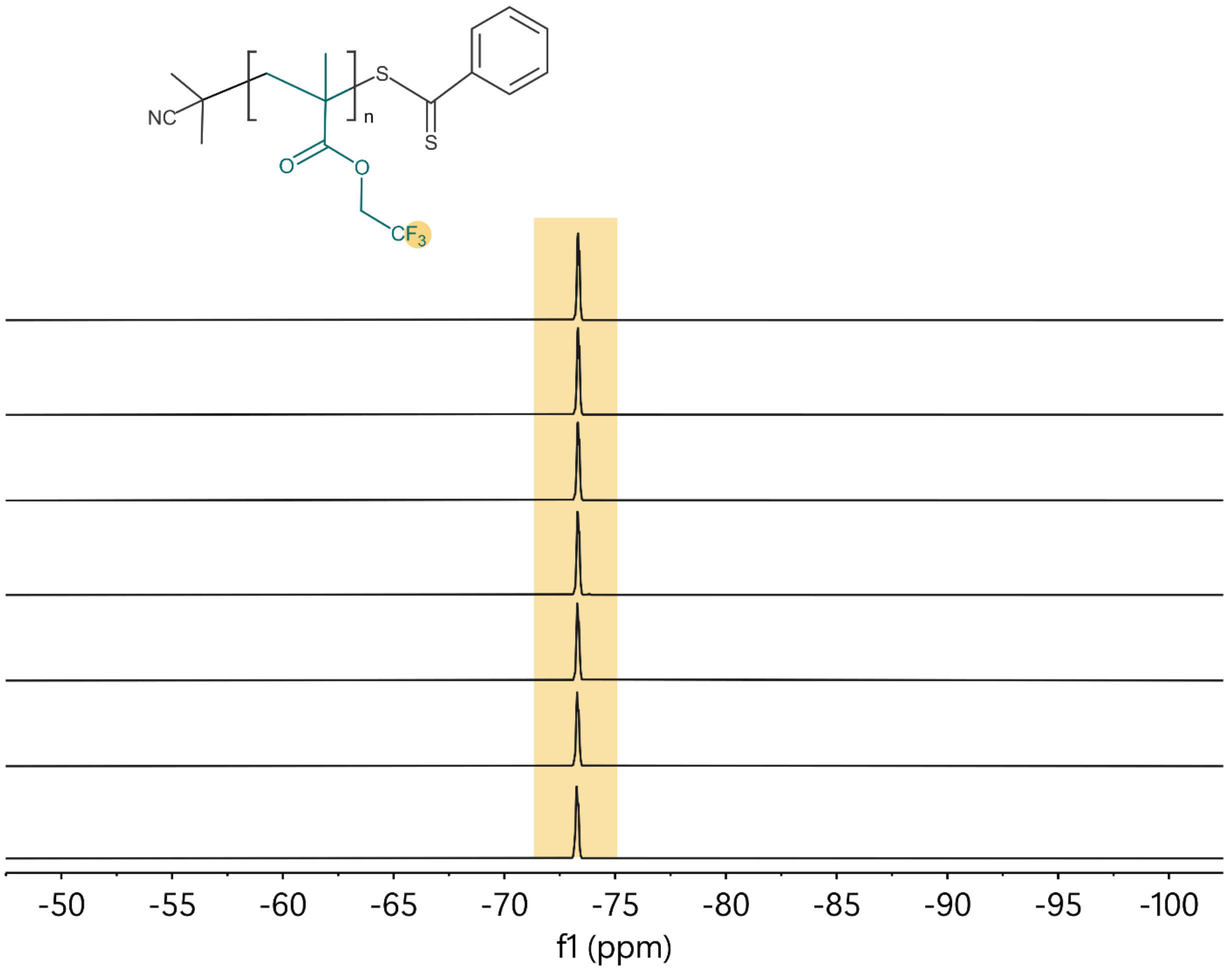

Figure S4. ^{19}F NMR (400 MHz, $CDCl_3$) spectra of PTFEMA macro-CTA. δ (ppm): -73.32 (–C**F**$_3$).[2,3]

Table S1. Number average molecular weight (M_n) and dispersity ($Đ$) of PTFEMA macro-CTA.

PTFEMA macro-CTA #	Targeted M_n (kg mol^{-1})	Reaction time (h)	TFEMA conversion [a] (%)	M_n [b] (kg mol^{-1})	$Đ$ [b]
1	10	1.00	---	5.20	1.17
2	10	1.00	13.42	5.40	1.17
3	10	1.00	---	5.70	1.17
4	10	4.00	---	7.30	1.20
5	10	4.00	48.59	7.50	1.19
6	10	6.00	65.03	9.00	1.15
7	10	8.00	73.79	10.10	1.14
8	15	6.00	61.24	10.85	1.21
9	25	3.90	36.51	11.10	1.24
10	25	5.00	---	12.10	1.23
11	25	8.72	50.98	13.10	1.23
12	35	4.00	31.27	13.40	1.24
13	25	10.38	57.98	15.00	1.22
14	25	12.02	65.22	15.30	1.20
15	25	11.00	---	15.70	1.19
16	35	8.00	54.02	16.70	1.23
17	25	16.00	72.11	16.80	1.19
18	35	12.33	65.10	19.80	1.21
19	35	15.00	72.14	20.50	1.20
20	35	18.00	75.00	21.00	1.20
21	45	18.00	79.68	23.30	1.21
22	50	20.33	65.52	24.70	1.24

[a] Conversion was calculated from the ^{1}H NMR peak integrals (I) of TFEMA (δ = 6.23 ppm and 5.70 ppm: 2H, $H_2C{=}C$<) and PTFEMA (δ = 4.34 ppm: *s*, 2H, $-CH_2-CF_3$) of the crude solutions according to the following expression: $I_{PTFEMA}/(I_{PTFEMA} + I_{TFEMA})$.

[b] Determined by SEC in THF using PS standards.

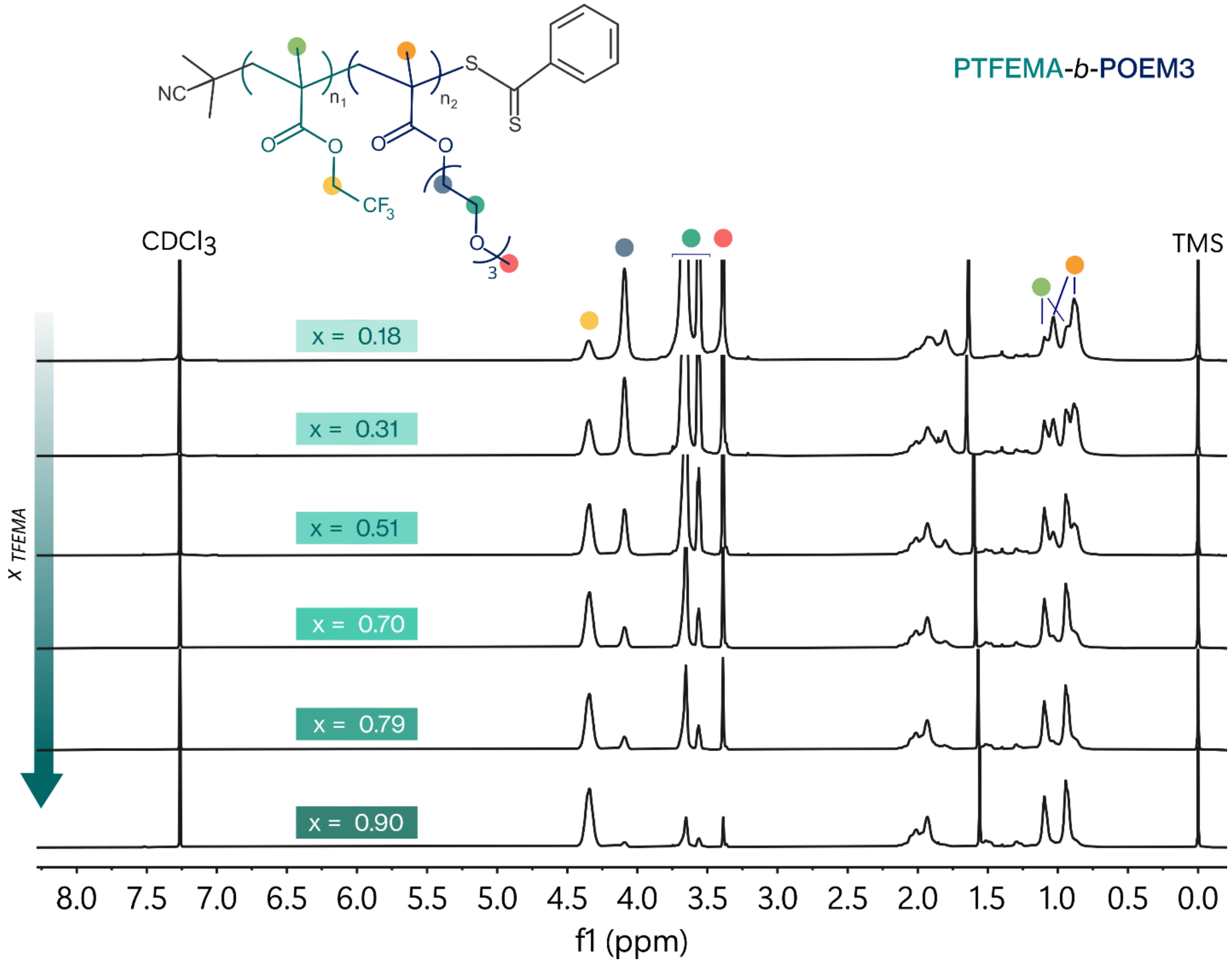

Figure S5. ^{1}H NMR (400 MHz, $CDCl_3$) spectra of PTFEMA-*b*-$POEM_3$. δ (ppm): 0.80–1.20 (*m*, 3H, –C**H**$_3$), 3.38 (*s*, 3H, −O−C**H**$_3$), 3.43−3.86 (*m*, 4H, −C**H**$_2$−C**H**$_2$−O−), 4.08 (*s*, 2H, −C(O)−O−C**H**$_2$−), 4.34 (*s*, 2H, -C**H**$_2$-CF_3).[4,5]

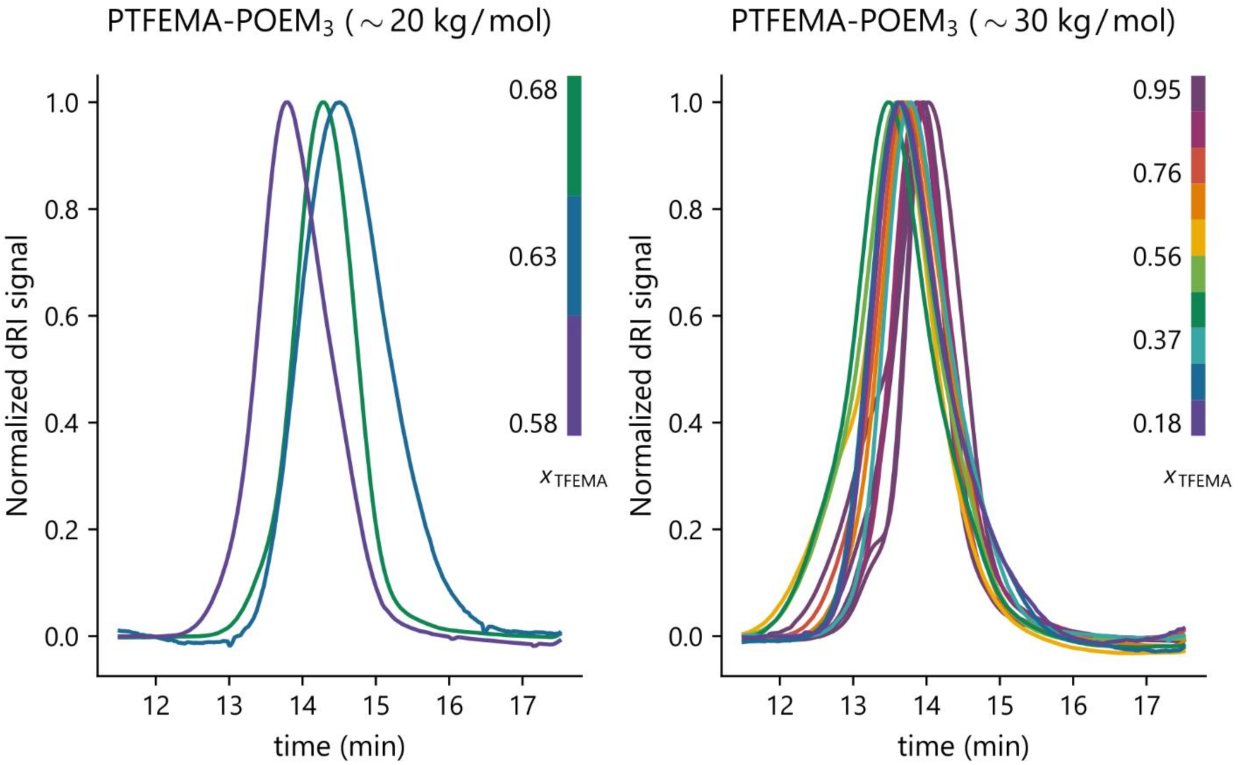

Figure S6. SEC traces of PTFEMA-*b*-POEM$_3$ copolymers series of ~20 kg mol^{-1} and 30 kg mol^{-1}.

Table S2. Reaction parameters, TFEMA mass fraction (x_{TFEMA}), number average molecular weight (M_n) and dispersity ($Đ$) of PTFEMA-*b*-$POEM_3$ copolymers.

PTFEMA–*b*–$POEM_3$ Entry #	PTFEMA M_n [a] (kg mol^{-1})	Reaction time (h)	x_{TFEMA} [b]	M_n [c] (kg mol^{-1})	$Đ$ [a]
1	5.2	10.63	0.18	28.9	1.15
2	9.0	9.58	0.31	29.0	1.15
3	13.1	8.67	0.35	37.4	1.28
4	11.1	8.67	0.41	27.1	1.26
5	12.1	8.67	0.43	28.1	1.19
6	13.4	8.17	0.51	26.3	1.20
7	15.7	6.78	0.55	28.5	1.20
8	10.1	4.00	0.58	17.4	1.17
9	16.7	6.00	0.60	27.8	1.21
10	13.4	5.17	0.63	21.3	1.28
11	16.7	5.50	0.64	26.1	1.21
12	16.7	4.83	0.68	24.6	1.21
13	20.5	4.83	0.70	29.3	1.33
14	16.8	3.90	0.74	22.7	1.19
15	23.3	3.50	0.76	30.7	1.33
16	24.7	3.00	0.79	31.3	1.33
17	20.5	2.33	0.81	25.3	1.21
18	24.7	2.33	0.90	27.4	1.26
19	24.7	1.67	0.95	26.0	1.27

[a] Determined by SEC in THF using PS standards.

[b] Calculated from the molar ratios obtained by ^{1}H NMR peak integrals (I) of PTFEMA (δ = 4.34 ppm: *s*, 2H, $-CH_2-CF_3$) and $POEM_3$ (δ = 4.08 ppm: *s*, 2H, $-C(O)-O-CH_2-$) of purified and dried block copolymer samples according to the following expression: $[I_{CH2(PTFEMA)} * M_{w\ TFEMA}]/[I_{CH2(PTFEMA)} * M_{w\ TFEMA} + I_{CH2(POEM3)} * M_{w\ OEM3}]$, where $M_{w\ TFEMA}$ and $M_{w\ OEM3}$ are the molar mass of TFEMA and OEM_3 units, 168.11 g mol^{-1} and 232.27 g mol^{-1}, respectively.

[c] Calculated from the molar ratios obtained by ^{1}H NMR and based on the M_n first calculated by SEC for PTFEMA.

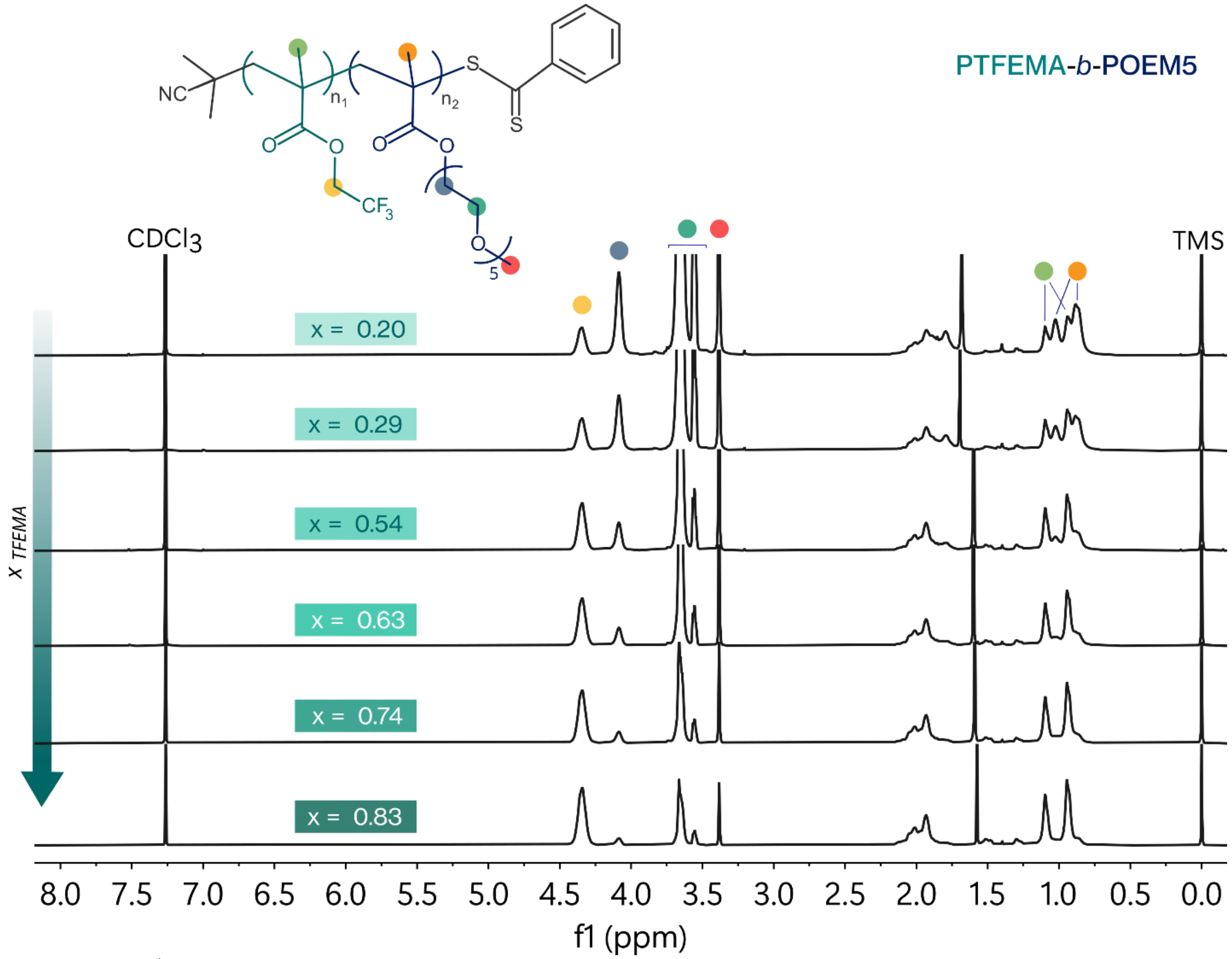

Figure S7. ^{1}H NMR (400 MHz, $CDCl_3$) spectra of PTFEMA-*b*-$POEM_5$. δ (ppm): 0.80–1.20 (*m*, 3H, –C**H**$_3$), 3.38 (*s*, 3H, −O−C**H**$_3$), 3.43−3.86 (*m*, 4H, −C**H**$_2$−C**H**$_2$−O−), 4.08 (*s*, 2H, −C(O)−O−C**H**$_2$−), 4.34 (*s*, 2H, –C**H**$_2$–CF_3).[4,5]

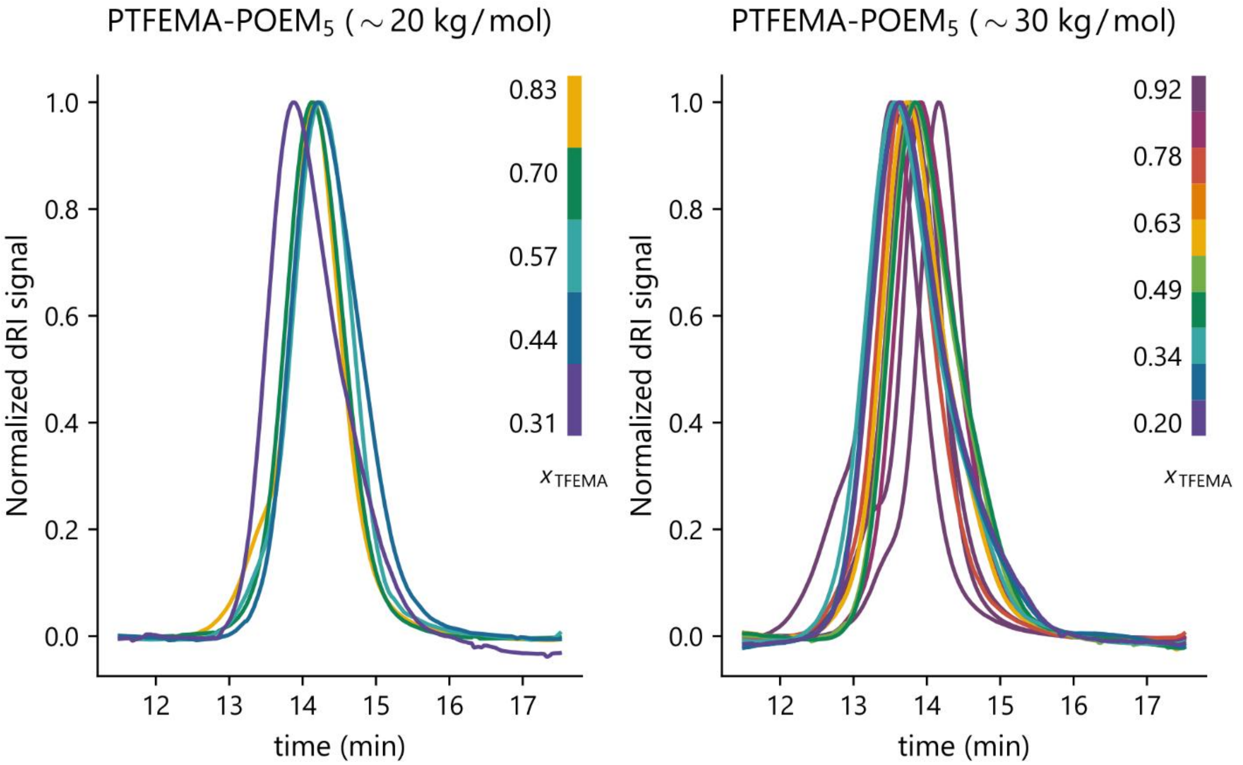

Figure S8. SEC traces of PTFEMA-*b*-POEM$_5$ copolymers series of ~20 kg mol^{-1} and 30 kg mol^{-1}.

Table S3. Reaction parameters, TFEMA mass fraction (x_{TFEMA}), number average molecular weight (M_n) and dispersity ($Đ$) of PTFEMA-*b*-POEM$_5$ copolymers.

PTFEMA–*b*–POEM$_5$ Entry #	PTFEMA M_n [a] (kg mol^{-1})	Reaction time (h)	x_{TFEMA} [b]	M_n [c] (kg mol^{-1})	$Đ$ [a]
1	5.2	5.00	0.20	26.0	1.15
2	9.0	4.75	0.29	31.0	1.16
3	7.3	3.67	0.31	23.5	1.19
4	11.1	3.83	0.46	24.1	1.14
5	16.8	4.00	0.51	32.9	1.22
6	10.1	4.25	0.54	18.7	1.17
7	15.7	3.60	0.61	25.7	1.18
8	16.8	3.83	0.63	26.7	1.19
9	15.0	2.33	0.68	22.1	1.19
10	19.8	3.00	0.72	27.5	1.20
11	20.5	3.00	0.74	27.7	1.20
12	19.8	2.42	0.77	25.7	1.19
13	19.8	2.00	0.79	25.1	1.19
14	24.7	3.45	0.79	31.3	1.27
15	19.8	2.00	0.83	23.9	1.20
16	24.7	0.83	0.91	27.1	1.27

[a] Determined by SEC in THF using PS standards.

[b] Calculated from the molar ratios obtained by ^{1}H NMR peak integrals (I) of PTFEMA (δ = 4.34 ppm: *s*, 2H, $-CH_2-CF_3$) and POEM$_5$ (δ = 4.08 ppm: *s*, 2H, $-C(O)-O-CH_2-$) of purified and dried block copolymer samples according to the following expression: $[I_{CH2(PTFEMA)} * M_{w\ TFEMA}]/[I_{CH2(PTFEMA)} * M_{w\ TFEMA} + I_{CH2(POEM5)} * M_{w\ OEM5}]$, where $M_{w\ TFEMA}$ and $M_{w\ OEM5}$ are the molar mass of TFEMA and OEM$_5$ units, 168.11 g mol^{-1} and 300 g mol^{-1}, respectively.

[c] Calculated from the molar ratios obtained by ^{1}H NMR and based on the M_n first calculated by SEC for PTFEMA.

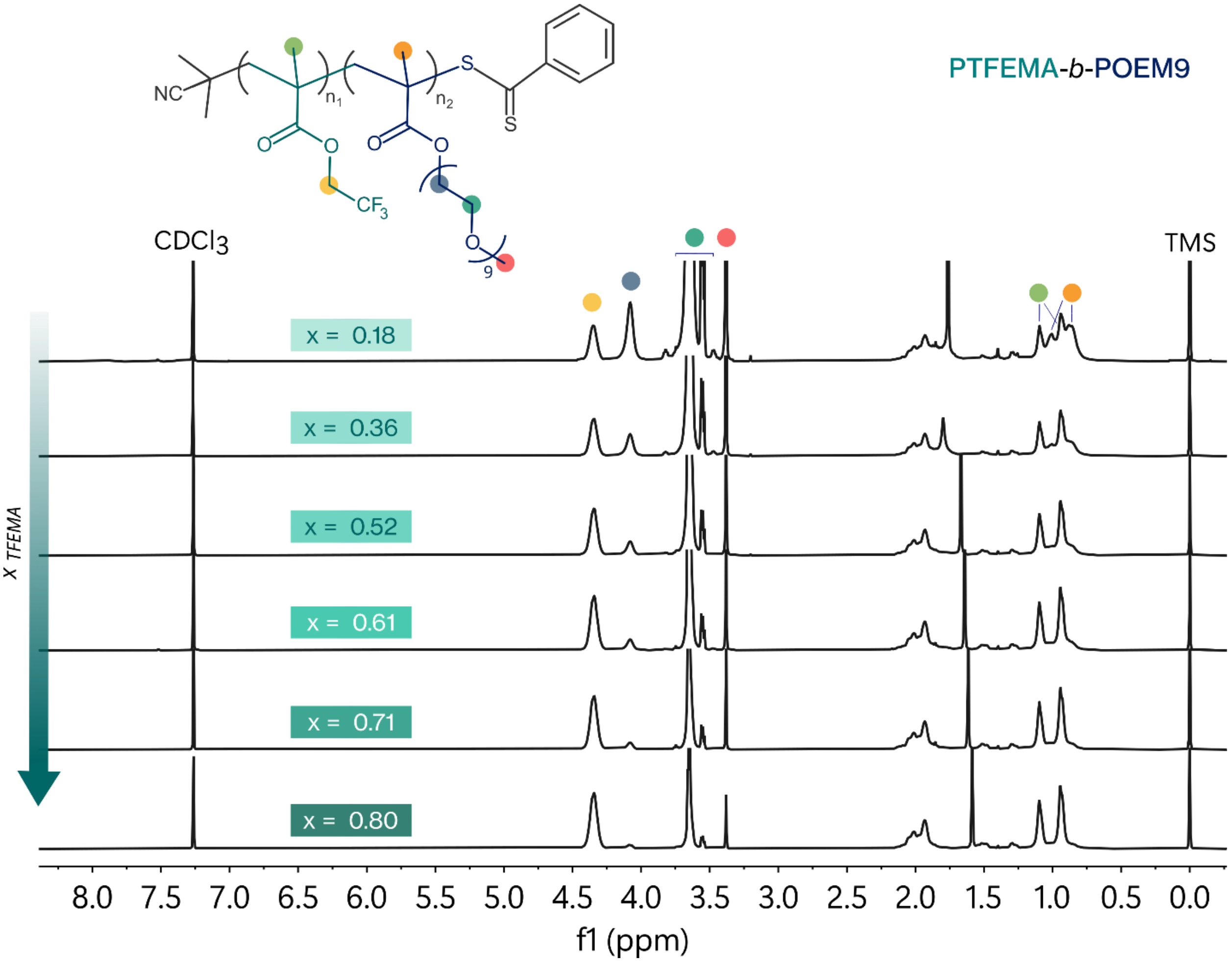

Figure S9. ^{1}H NMR (400 MHz, $CDCl_3$) spectra of PTFEMA-*b*-POEM$_9$. δ (ppm): 0.80–1.20 (*d*, 3H, –C**H**$_3$), 3.38 (*s*, 3H, –O–C**H**$_3$), 3.43–3.86 (*m*, 4H, –C**H**$_2$–C**H**$_2$–O–), 4.08 (*s*, 2H, –C(O)–O–C**H**$_2$–), 4.34 (*s*, 2H, –C**H**$_2$–CF_3).[4,5]

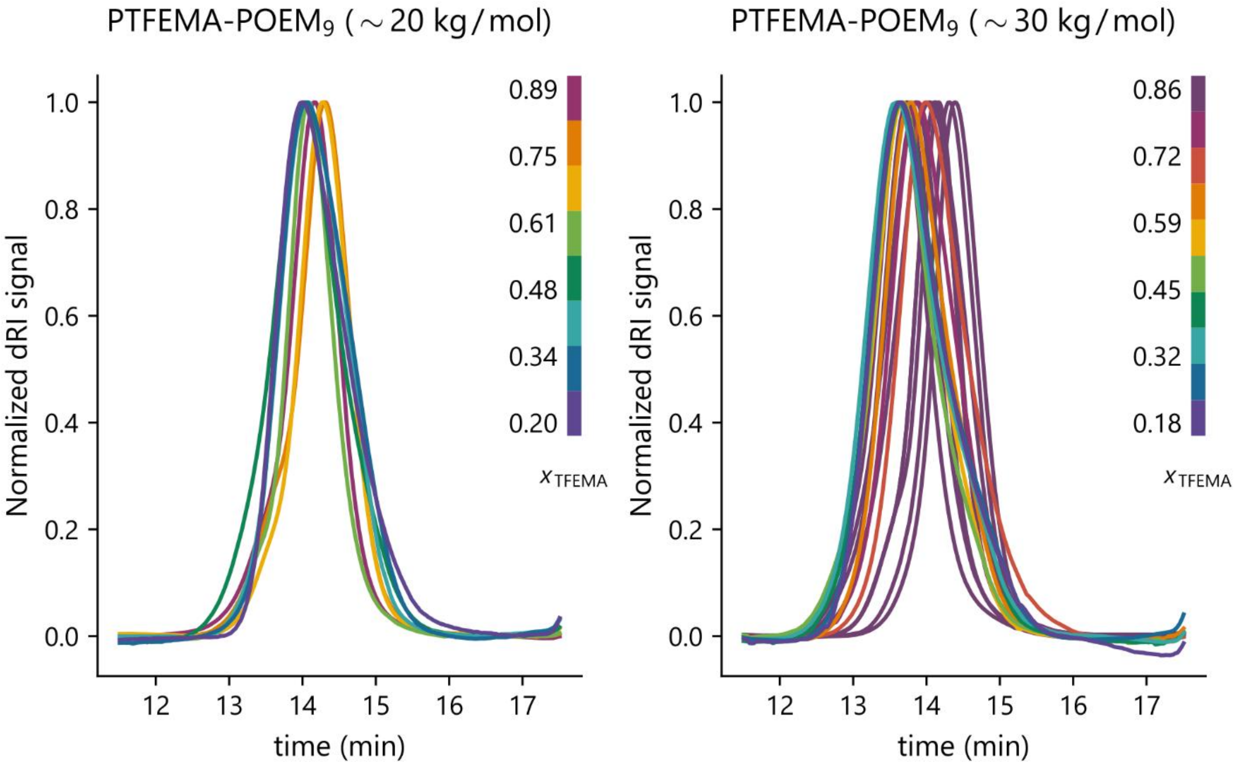

Figure S10. SEC traces of PTFEMA-*b*-$POEM_9$ copolymers series of ~20 kg mol^{-1} and 30 kg mol^{-1}.

Table S4. Reaction parameters, TFEMA mass fraction (x_{TFEMA}), number average molecular weight (M_n) and dispersity ($Đ$) of PTFEMA-*b*-POEM$_9$ copolymers.

PTFEMA–*b*–POEM$_9$ Entry #	PTFEMA macro-CTA M_n [a] (kg mol^{-1})	Reaction time (h)	x_{TFEMA} [b]	M_n [c] (kg mol^{-1})	$Đ$ [a]
1	5.4	6.20	0.18	30.0	1.14
2	5.7	3.00	0.20	28.5	1.16
3	7.5	5.08	0.26	28.8	1.13
4	5.7	2.25	0.28	20.4	1.17
5	7.5	3.75	0.28	26.8	1.11
6	7.5	2.40	0.32	23.4	1.13
7	10.9	5.00	0.36	30.3	1.18
8	10.1	2.50	0.38	26.6	1.12
9	10.1	3.07	0.43	23.5	1.13
10	13.4	2.75	0.43	31.2	1.18
11	15.3	3.25	0.45	34.0	1.16
12	15.7	2.63	0.52	30.2	1.16
13	13.4	2.28	0.54	24.8	1.19
14	10.9	4.87	0.55	19.8	1.23
15	16.7	2.33	0.55	30.4	1.19
16	19.8	3.07	0.61	32.5	1.22
17	13.1	1.30	0.62	21.1	1.17
18	19.8	1.93	0.68	29.1	1.18
19	15.7	1.17	0.75	20.9	1.20
20	19.8	1.45	0.71	27.9	1.21
21	21.0	1.17	0.75	28.0	1.22
22	15.0	0.58	0.78	19.2	1.18
23	23.3	1.50	0.80	29.1	1.26
24	23.3	1.00	0.85	27.4	1.24
25	23.3	1.23	0.86	27.1	1.24

[a] Determined by SEC in THF using PS standards.

[b] Calculated from the molar ratios obtained by ^{1}H NMR peak integrals (I) of PTFEMA (δ = 4.34 ppm: *s*, 2H, $-CH_2-CF_3$) and POEM$_9$ (δ = 4.08 ppm: *s*, 2H, $-C(O)-O-CH_2-$) of purified and dried block copolymer samples according to the following expression: $[I_{CH2(PTFEMA)} * M_{w\,TFEMA}]/[I_{CH2(PTFEMA)} * M_{w\,TFEMA} + I_{CH2(POEM9)} * M_{w\,OEM9}]$, where $M_{w\,TFEMA}$ and $M_{w\,OEM9}$ are the molar mass of TFEMA and OEM$_9$ units, 168.11 g mol^{-1} and 500 g mol^{-1}, respectively.

[c] Calculated from the molar ratios obtained by ^{1}H NMR and based on the M_n first calculated by SEC for PTFEMA.

Density estimation by the van Krevelen group contribution method

Densities for the POEM$_x$ segments were estimated from:

$$\rho\,(298\ \mathrm{K}) = \frac{M}{V_r\,(298\ \mathrm{K})}$$

where M is the average molecular weight of the repeat unit (232.27 g mol^{-1}, g mol^{-1}, 300 g mol^{-1} and 500 g mol^{-1} for OEM$_3$, OEM$_5$ and OEM$_9$, respectively) and V_r is the standard molecular volume at room temperature for a rubbery amorphous polymer. The V_r values for each polymer were predicted from the expression below by using the Krevelen group contributions values listed in Table S5.[6,7]

$$V_r(298\ \mathrm{K}) = \sum_i V_i\,(298\ \mathrm{K})$$

The estimated density values for POEM$_3$, POEM$_5$ and POEM$_9$ were 1.17 g cm^{-3}, 1.16 g cm^{-3} and 1.13 g cm^{-3}, respectively.

Table S5. Standard molecular volume of POEM$_x$ determined using Krevelen group contribution.

**Group**	**V_i (289) (cm^3/mol)**	**V_i (289) POEM$_3$**	**V_i (289) POEM$_5$***	**V_i (289) POEM$_9$**	**POEM$_x$**
**$-CH_3$**	23	23 (2)	23 (2)	23 (2)	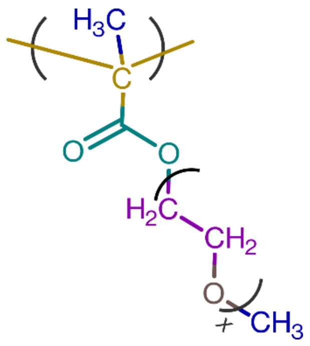
**> C <**	5.32	5.32 (1)	5.32 (1)	5.32 (1)	
**−COO−**	24.60	24.60 (1)	24.60 (1)	24.60 (1)	
**$-CH_2-$**	16.37	16.37 (6)	16.37 (9)	16.37 (18)	
**−O−**	8	8 (3)	8 (4.5)	8 (9)	
**V_r(298 K) (cm^3/mol)**		**198.14**	**259.25**	**442.58**	

*V_r was predicted considering 4.5 EO units in the repeat unit; for practical reasons we referred to this monomer as OEM$_5$ through all the text.

Parameters setting for SCFT from experimental data

The total degree of polymerization was rescaled as input for the SCFT (denoted as N_{Total}) according to the following expressions:

$$N_{Total} = N_{PTFEMA} + N_{POEMx}$$

$$N_{PTFEMA} = \frac{V_{PTFEMA}}{v_{ref}} \qquad N_{POEMx} = \frac{V_{POEMx}}{v_{ref}}$$

where v_{ref} is the reference volume (118 Å^3), and V is the segment molecular volume defined as:

$$V = N' \times v_0$$

$$N' = \frac{M}{m_0} \qquad v_0 = \frac{m_0}{N_{Av}\rho}$$

where *N'* is the number of repeat units in the segment, *M* is the segment number average molecular weight, m_0 is the molecular weight of the corresponding repeat unit (168.11 g mol^{-1}, 232.27 g mol^{-1}, g mol^{-1}, 300 g mol^{-1} and 500 g mol^{-1} for TFEMA, OEM$_3$, OEM$_5$ and OEM$_9$, respectively), ρ is the segment density (1.45 g cm^{-3}, 1.17 g cm^{-3}, 1.16 g cm^{-3}, 1.13 g cm^{-3} for PTFEMA, POEM$_3$, POEM$_5$ and POEM$_9$, respectively), and N_{Av} is the Avogadro's number.

Volume fractions (*f*) were calculated from the values obtained above as:

$$f_{PTFEMA} = \frac{N_{PTFEMA}}{N_{PTFEMA} + N_{POEMx}}$$

$$f_{POEMx} = 1 - f_{PTFEMA}$$

Table S6. Characteristics of PTFEMA-*b*-**POEM**$_3$ copolymers.

Entry #	x_{PTFEMA} [a]	x_{POEM3} [a]	N_{PTFEMA} [b]	N_{POEM3} [b]	N_{Total} [b]	f_{POEM3} [b]	Morphology [c]
1	0.18	0.82	50	285	335	0.85	DIS
2	0.31	0.69	87	241	328	0.73	HEX
3	0.35	0.65	127	293	420	0.70	HEX
4	0.41	0.59	108	192	300	0.64	HEX
5	0.43	0.57	117	193	310	0.62	HEX
6	0.51	0.49	130	155	285	0.54	HEX+LAM
7	0.55	0.45	152	155	307	0.50	LAM
8	0.58	0.42	98	88	186	0.47	LAM
9	0.60	0.40	162	134	296	0.45	LAM
10	0.63	0.37	130	95	225	0.42	LAM
11	0.64	0.36	162	113	275	0.41	LAM
12	0.68	0.32	162	95	257	0.37	LAM
13	0.70	0.30	199	106	305	0.35	LAM
14	0.74	0.26	163	71	234	0.30	HEX
15	0.76	0.24	226	88	315	0.28	HEX+LAM
16	0.79	0.21	240	79	319	0.25	HEX
17	0.81	0.19	199	58	257	0.23	HEX
18	0.90	0.10	240	33	273	0.12	DIS
19	0.95	0.05	240	16	255	0.06	DIS

[a] Calculated from the molar ratio of both blocks determined by ^{1}H NMR.

[b] Calculated using a 118 Å^3 reference volume and densities of ρ(PTFEMA) = 1.45 g/cm^3 and ρ(POEM$_3$) = 1.17 g/cm^3 (25 °C).

[c] Determined by SAXS.

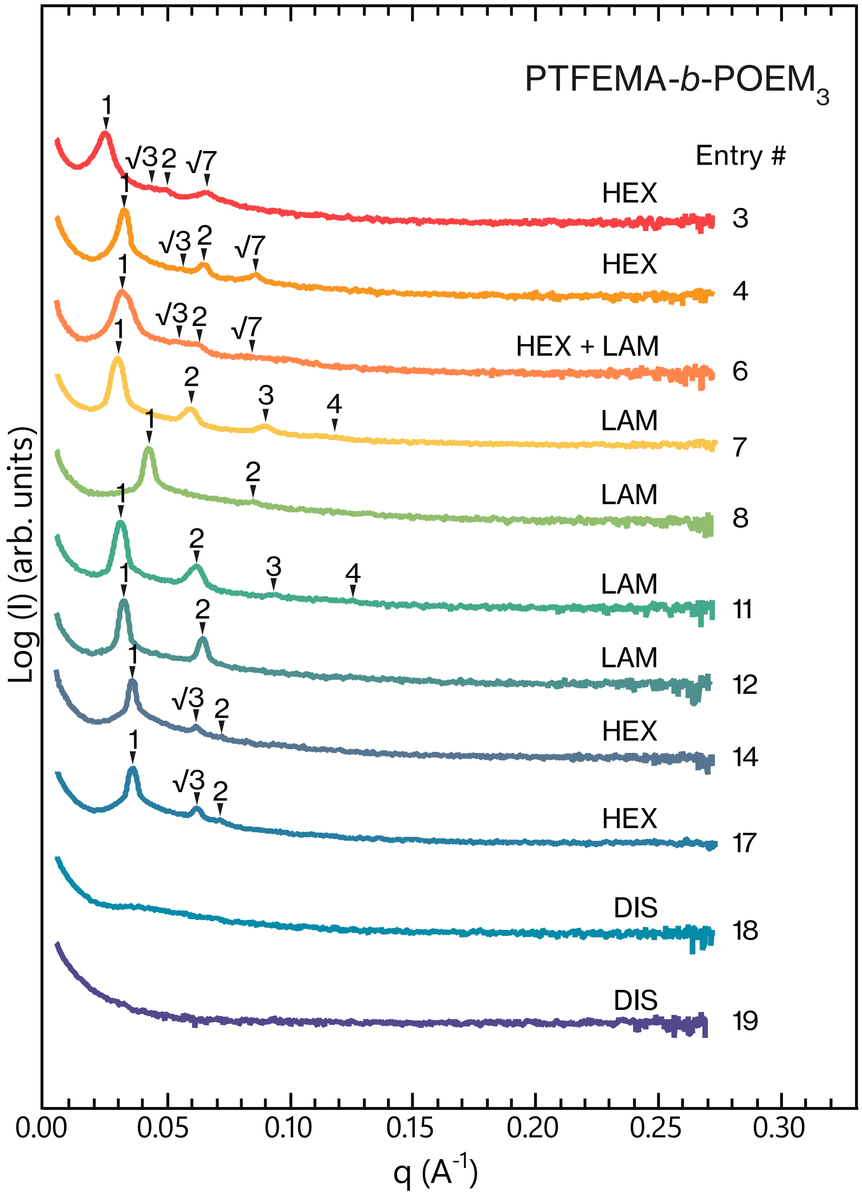

Figure S11. Room temperature SAXS patterns for PTFEMA-*b*-POEM3 copolymers. DIS, HEX, and LAM denote disordered, hexagonal, and lamellar phase, respectively.

Table S7. Characteristics of PTFEMA-*b*-**POEM**$_5$ copolymers.

**Entry #**	x_{PTFEMA} [a]	x_{POEM5} [a]	N_{PTFEMA} [b]	N_{POEM5} [b]	N_{Total} [b]	f_{POEM5} [b]	**Morphology** [c]
1	0.20	0.80	50	252	303	0.83	DIS
2	0.29	0.71	87	267	355	0.75	HEX
3	0.31	0.69	71	197	268	0.74	DIS
4	0.46	0.54	108	158	266	0.59	HEX
5	0.51	0.49	163	196	359	0.55	HEX
6	0.54	0.46	98	104	202	0.52	LAM
7	0.61	0.39	152	122	274	0.44	LAM
8	0.63	0.37	163	120	283	0.42	LAM
9	0.68	0.32	146	86	231	0.37	LAM
10	0.72	0.28	192	93	286	0.33	LAM
11	0.74	0.26	199	87	286	0.31	LAM+HEX
12	0.77	0.23	192	72	264	0.27	HEX
13	0.79	0.21	192	64	256	0.25	HEX
14	0.79	0.21	240	80	319	0.25	HEX+GYR
15	0.83	0.17	192	49	241	0.20	DIS
16	0.91	0.09	240	30	269	0.11	DIS

[a] Calculated from the molar ratio of both blocks determined by ^1H NMR.

[b] Calculated using a 118 $Å^3$ reference volume and densities of ρ(PTFEMA) = 1.45 g/cm^3 and ρ($POEM_5$) = 1.16 g/cm^3 (25 °C).

[c] Determined by SAXS.

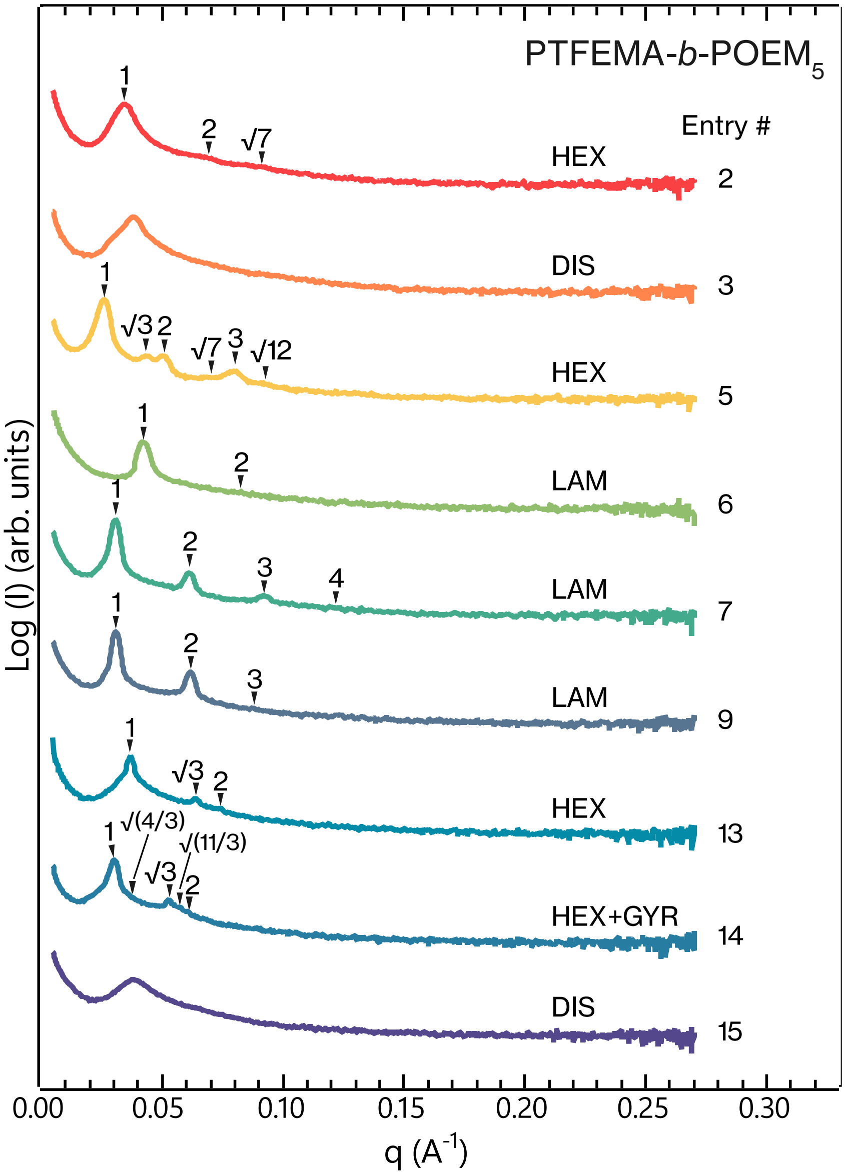

Figure S12. Room temperature SAXS patterns for PTFEMA-*b*-POEM$_5$ copolymers. DIS, HEX, GYR and LAM denote disordered, hexagonal, gyroid and lamellar phase, respectively.

Note: In Figure S12 - Entry #14, the (200) diffraction peak associated with the gyroid phase and characterized by $\frac{q}{q^*} = \sqrt{(4/3)}$, exhibits very low intensity in the corresponding SAXS pattern. This peak appears as a shoulder within the primary peak, making it difficult to assert with absolute certainty the presence of the gyroid phase in the PTFEMA-POEM$_5$ phase diagram.

Table S8. Characteristics of PTFEMA-*b*-**POEM**$_9$ copolymers.

**Entry #**	x_{PTFEMA} [a]	x_{POEM9} [a]	N_{PTFEMA} [b]	N_{POEM9} [b]	N_{Total} [b]	f_{POEM9} [b]	**Morphology** [c]
1	0.18	0.82	52	306	359	0.85	DIS
2	0.20	0.80	55	284	339	0.84	DIS
3	0.26	0.74	73	266	339	0.79	DIS
4	0.28	0.72	55	183	238	0.77	DIS
5	0.28	0.72	73	240	313	0.77	DIS
6	0.32	0.68	73	198	271	0.73	DIS
7	0.36	0.64	106	241	347	0.70	HEX
8	0.38	0.62	98	205	303	0.68	HEX
9	0.43	0.57	98	167	265	0.63	HEX
10	0.43	0.57	130	221	351	0.63	HEX
11	0.45	0.55	148	233	381	0.61	HEX
12	0.52	0.48	152	180	333	0.54	HEX
13	0.54	0.46	130	142	272	0.52	HEX
14	0.55	0.45	106	111	217	0.51	HEX
15	0.55	0.45	162	170	332	0.51	HEX+LAM
16	0.61	0.39	192	158	350	0.45	LAM
17	0.62	0.38	127	100	227	0.44	LAM
18	0.68	0.32	192	116	308	0.38	LAM
19	0.75	0.25	152	65	218	0.30	LAM
20	0.71	0.29	192	101	293	0.34	LAM
21	0.75	0.25	204	87	291	0.30	LAM
22	0.78	0.22	146	53	198	0.27	DIS
23	0.80	0.20	226	73	299	0.24	LAM
24	0.85	0.15	226	51	277	0.18	DIS
25	0.86	0.14	226	47	273	0.17	DIS

[a] Calculated from the molar ratio of both blocks determined by ^{1}H NMR.

[b] Calculated using a 118 Å^3 reference volume and densities of ρ(PTFEMA) = 1.45 g/cm^3 and ρ($POEM_9$) = 1.13 g/cm^3 (25 °C).

[c] Determined by SAXS

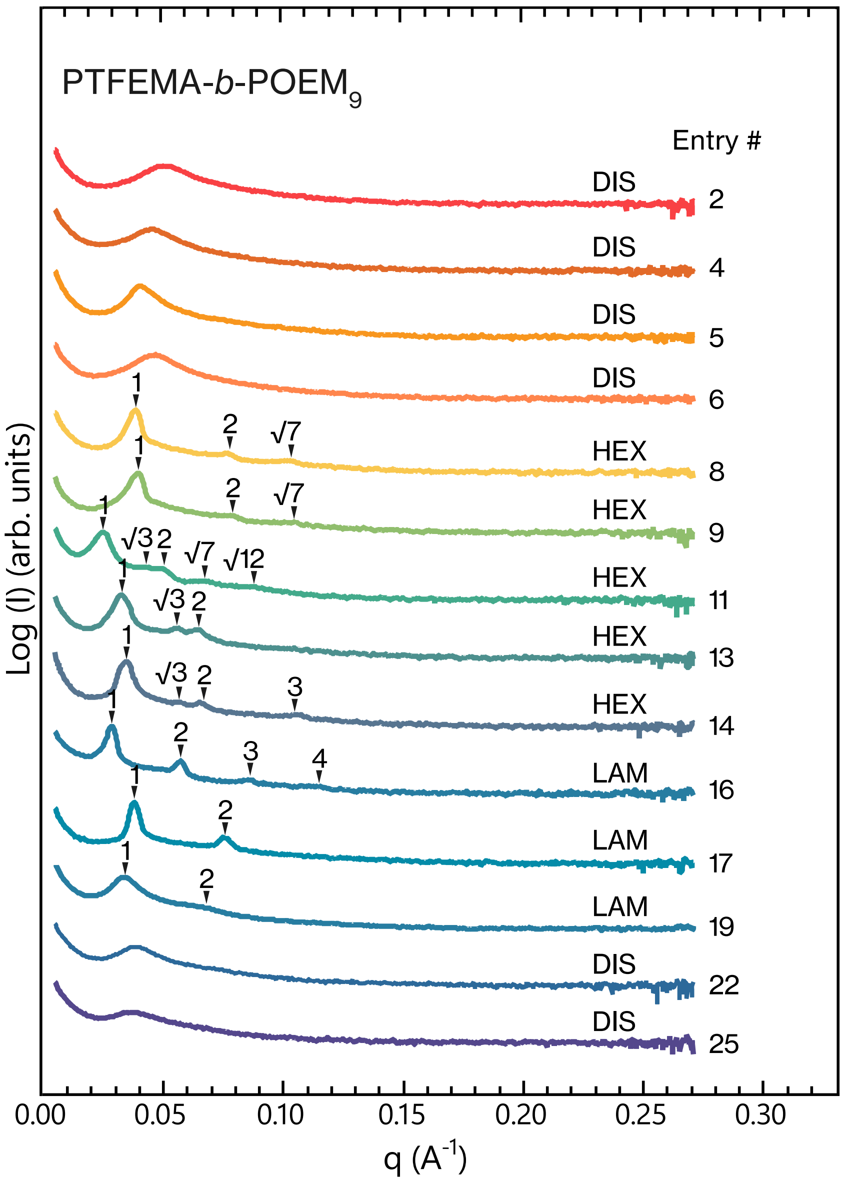

Figure S13. Room temperature SAXS patterns for PTFEMA-*b*-$POEM_9$ copolymers. DIS, HEX, and LAM denote disordered, hexagonal, and lamellar phase, respectively.

Table S9. Simulation systems listed by side-chain length and minimum and maximum f_B via bottlebrush scaling.

n_B	ν_B	$\varepsilon^{1/2}\eta_B/\eta_A$	$f_{B\,min}$	$f_{B\,max}$
1	2.0	1.050	0.13	0.93
2	3.0	1.249	0.20	0.90
3	4.0	1.382	0.20	0.90
5	6.0	1.570	0.20	0.85
7	8.0	1.708	0.20	0.82
9	10.0	1.819	0.20	0.75
11	11.0	1.912	0.50	0.90

Table S10. Simulation systems listed by side-chain length and minimum and maximum f_B via comb scaling.

n_B	ν_B	$\varepsilon^{1/2}\eta_B/\eta_A$	$f_{B\,min}$	$f_{B\,max}$
1	2.0	1.131	0.13	0.93
2	3.0	1.386	0.20	0.90
3	4.0	1.600	0.20	0.90
5	6.0	1.960	0.20	0.85
7	8.0	2.263	0.20	0.82
9	10.0	2.530	0.20	0.75
11	11.0	2.771	0.50	0.90

References

(1) Xu, Y.; Wang, W.; Wang, Y.; Zhu, J.; Uhrig, D.; Lu, X.; Keum, J. K.; Mays, J. W.; Hong, K. Fluorinated Bottlebrush Polymers Based on Poly(Trifluoroethyl Methacrylate): Synthesis and Characterization. *Polym Chem* **2016**, *7* (3), 680–688.

(2) György, C.; Derry, M. J.; Cornel, E. J.; Armes, S. P. Synthesis of Highly Transparent Diblock Copolymer Vesicles via RAFT Dispersion Polymerization of 2,2,2-Trifluoroethyl Methacrylate in n-Alkanes. *Macromolecules* **2021**, *54* (3), 1159–1169.

(3) Fan, M.; Alghassab, T. S.; Twyman, L. J. Increased Oxygen Solubility in Aqueous Media Using PEG-Poly- 2,2,2-Trifluoroethyl Methacrylate Copolymer Micelles and Their Potential Application as Volume Expanders and as an Artificial Blood Product. *ACS Appl Bio Mater* **2018**, *1* (3), 708–713.

(4) Ali, M. M.; Stöver, H. D. H. Well-Defined Amphiphilic Thermosensitive Copolymers Based on Poly(Ethylene Glycol Monomethacrylate) and Methyl Methacrylate Prepared by Atom Transfer Radical Polymerization. *Macromolecules* **2004**, *37* (14), 5219–5227.

(5) Zhang, C.; Peng, H.; Whittaker, A. K. NMR Investigation of Effect of Dissolved Salts on the Thermoresponsive Behavior of Oligo(Ethylene Glycol)-Methacrylate-Based Polymers. *J Polym Sci A Polym Chem* **2014**, *52* (16), 2375–2385.

(6) Van Krevelen, D. W.; Hoftyzer, P. J. Prediction of Polymer Densities. *J Appl Polym Sci* **1969**, *13* (5), 871–881.

(7) Van Krevelen, D. W. *Properties of Polymers: Their Correlation with Chemical Structure; Their Numerical Estimation and Prediction from Additive Group Contributions: Fourth Edition*; Elsevier, 2009.
